# Supplementary material for: Variable Outcomes in Neural Differentiation of Human PSCs Arise from Intrinsic Differences in Developmental Signaling Pathways
Source: Cell Rep. 2020 Jun 9;31(10):107732. doi: 10.1016/j.celrep.2020.107732 (PMC7296348; doi:10.1016/j.celrep.2020.107732)
Supplement: Document S1. Figures S1–S7 [file mmc1.pdf]

Cell Reports, Volume *31*

Supplemental Information

Variable Outcomes in Neural Differentiation of Human PSCs Arise from Intrinsic Differences in Developmental Signaling Pathways

Alessio Strano, Eleanor Tuck, Victoria E. Stubbs, and Frederick J. Livesey

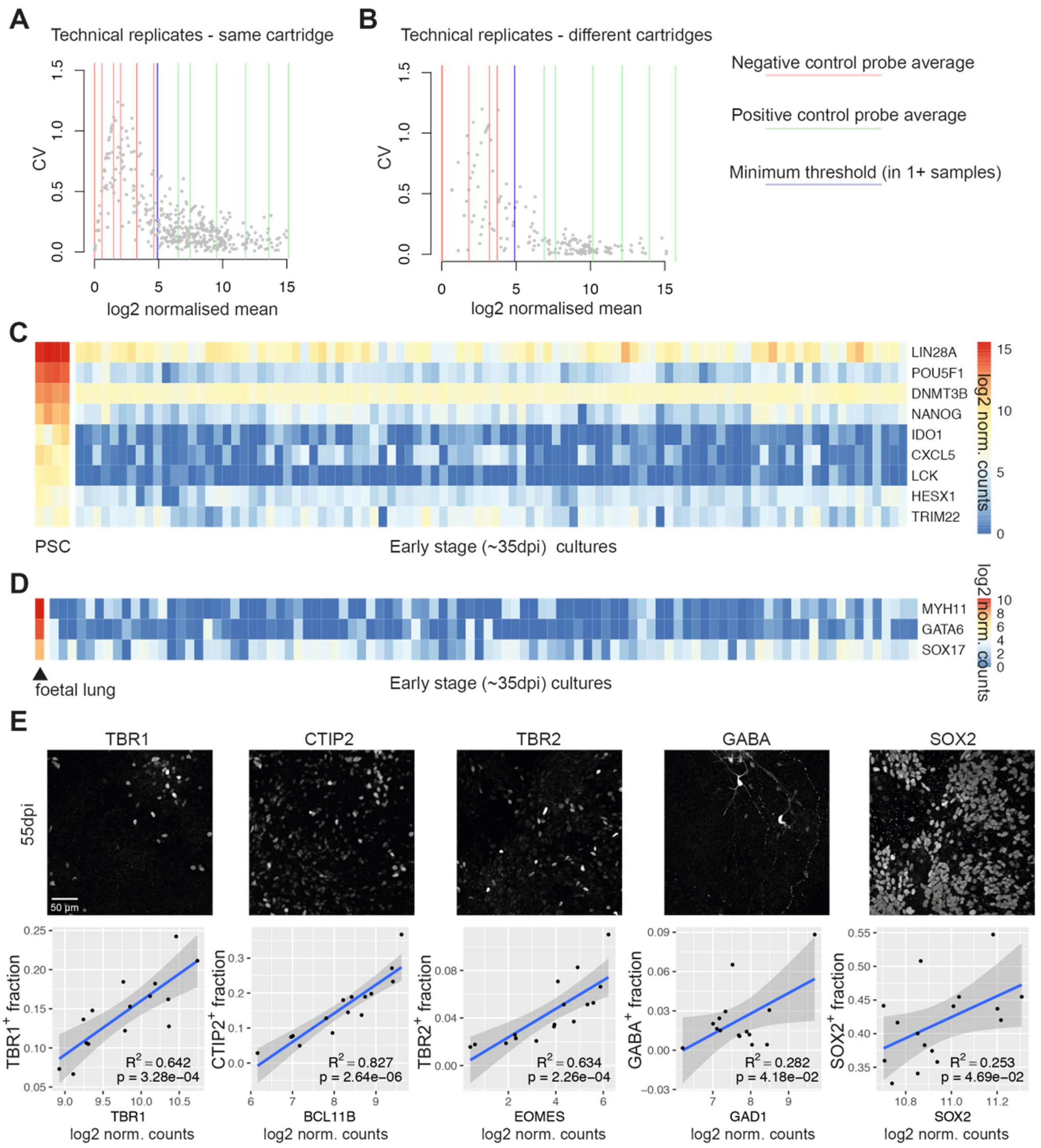

Figure S1. **Gene expression panel technical and biological validation.** Related to Figure 1.

(A-B) Relationship between mean gene expression and coefficient of variation in 3 technical replicates (3 samples each) run on the same cartridge (A) and 2 technical replicates run on different cartridges and 9 months apart (B). Dots represent individual gene probes; red and green vertical lines represent respectively the average for the negative and positive control probes included in the codeset. The blue vertical line represents the minimum threshold of expression we required in at least one sample to include a gene probe in any analysis.

(C) Heatmap of expression of PSC genes showing highly reduced or absent expression in 84 early-stage cortical differentiations compared to 4 PSC samples.

(D) Heatmap of expression of genes expressed in mesodermal and endodermal tissue showing no expression in 84 early-stage cortical differentiations compared to a 20pcw human foetal lung sample. (E) Correlations between expression of selected genes and the fraction of cells immunopositive for the corresponding protein (TBR1, CTIP2, TBR2, SOX2) or enzymatic product (GABA).

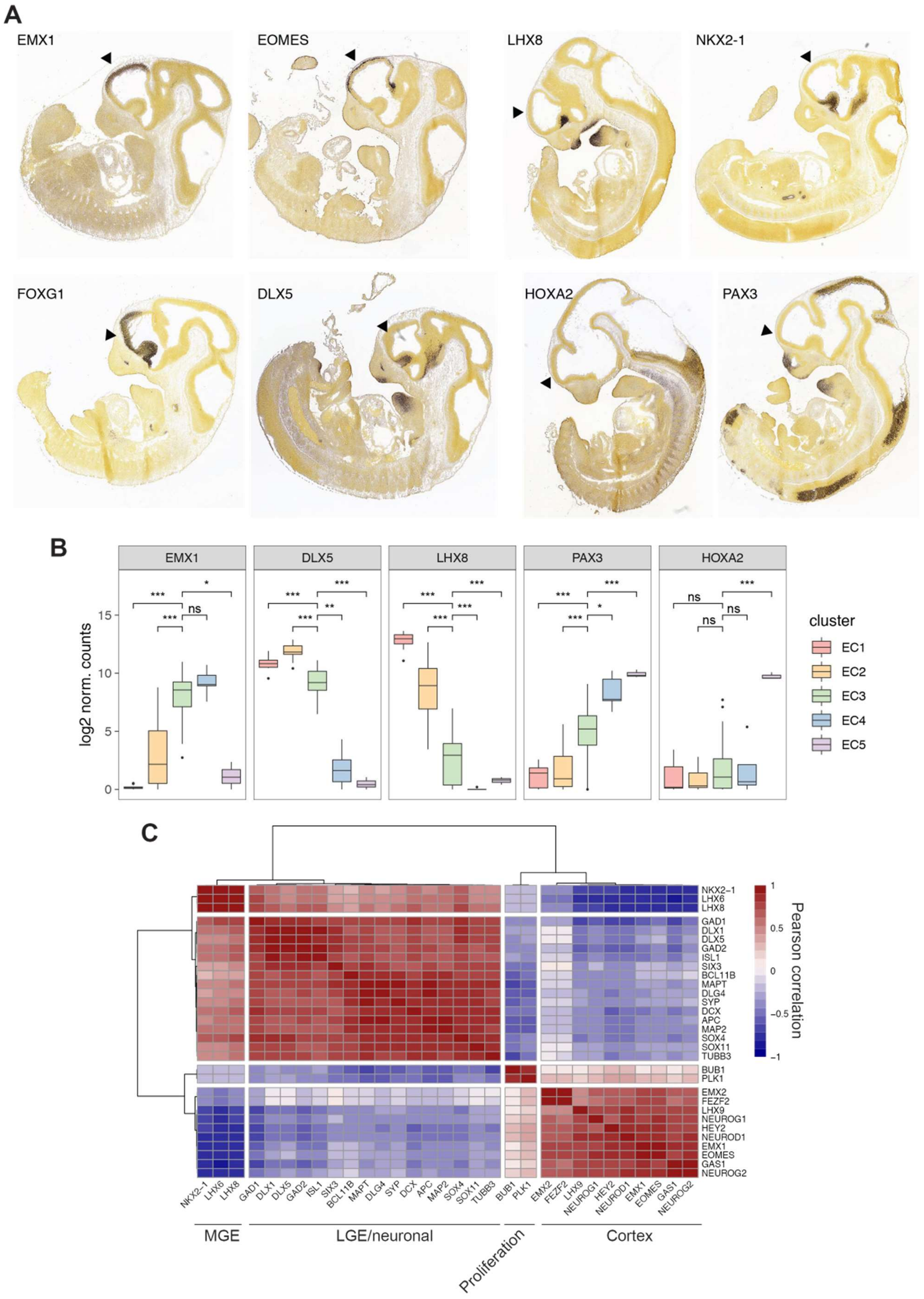
A
EMX1
EOMES
LHX8
NKX2-1
FOXG1
DLX5
HOXA2
PAX3
B
EMX1
DLX5
LHX8
PAX3
HOXA2
log2 norm. counts
cluster
EC1
EC2
EC3
EC4
EC5
C
Pearson correlation
MGE
LGE/neuronal
Proliferation
Cortex

Figure S2. Largest contributors to early-stage variation are enriched in specific brain regions during embryonic development. Related to Figure 2.

(A) Expression of selected high-loading genes measured by in-situ hybridisation in E11.5 mouse embryos (from the Allen Developing Mouse Brain Atlas © 2008 Allen Institute for Brain Science). A black arrowhead marks the developing cortex.

(B) Differences in expression of selected high-loading genes among the groups determined in Figure 2A (pairwise Welch's t-test, FDR corrected p-values: <0.05 (*), <0.01 (**), <0.001 (***), ns (not significant); only comparisons to EC3 are shown). Differentiations per group: EC1 (10), EC2 (28), EC3 (38), EC4 (5), EC5 (3).

(C) Heatmap of Pearson correlation coefficients for early-stage gene expression (n =84). Only genes with an absolute correlation coefficient greater than 0.85 for at least one other gene are included.

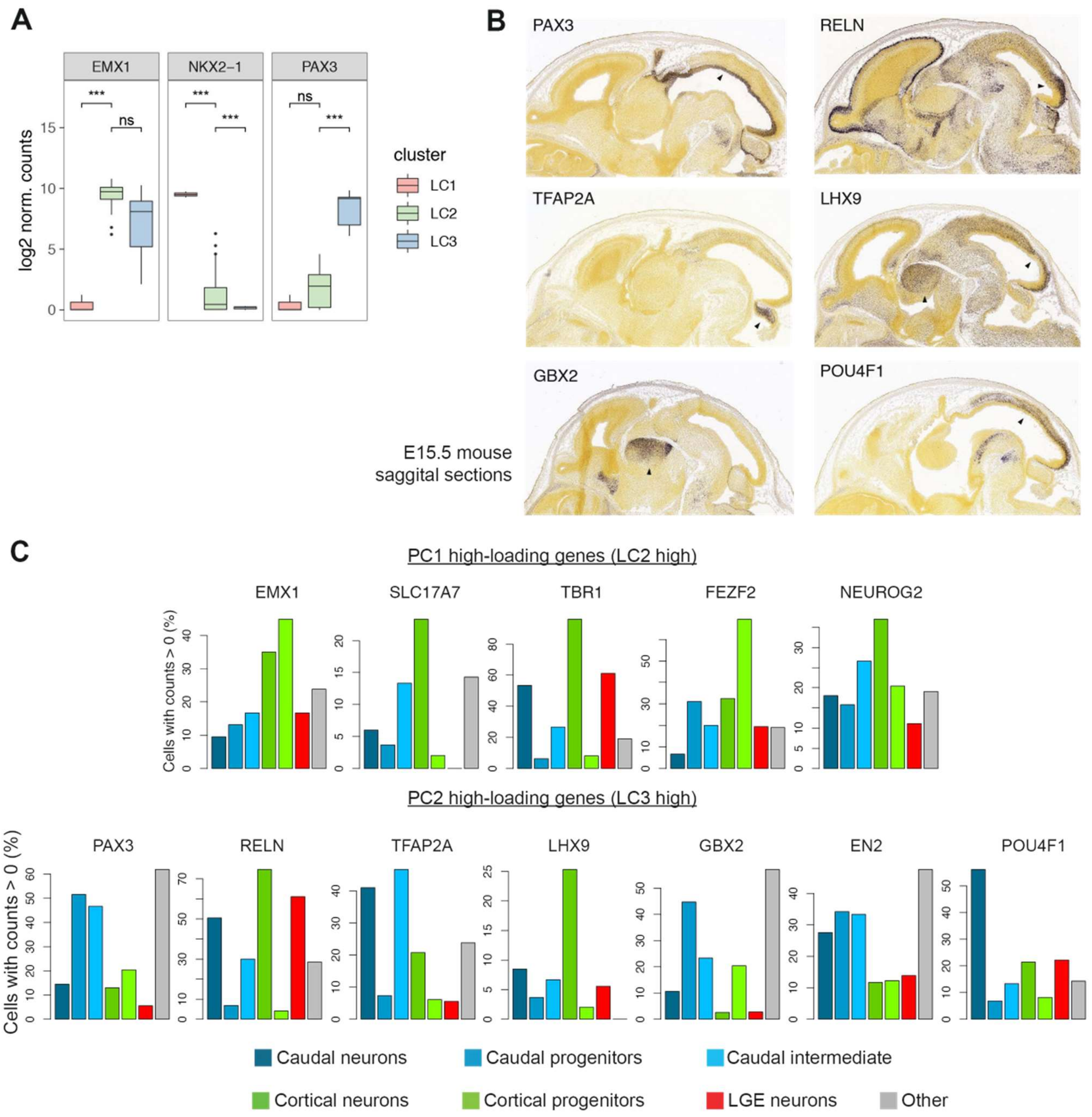

Figure S3. **Largest contributors to late-stage variation along PC2 are enriched in cell types with caudal identity.** Related to Figure 3.

(A) Differences in expression of selected high-loading genes among the groups determined in Figure 3A (pairwise Welch's t-test, FDR corrected p-values: <0.05 (*), <0.01 (**), <0.001 (***), ns (not significant)). Differentiations per group: LC1 (3), LC2 (36), LC3 (5).

(B) Expression of selected high-loading genes measured by in-situ hybridisation in E15.5 mouse embryos (from the Allen Developing Mouse Brain Atlas © 2008 Allen Institute for Brain Science).

(C) Expression of selected high-loading genes in scRNA-seq data from 54-dpi forebrain differentiations from Yao et al., 2016. For each gene, the percentage of single cells with at least one transcript count is plotted for cell populations of different lineages. Genes associated with LC1 are more highly expressed in progenitors and neurons of cortical lineage,

while most genes associated with LC2 are highly expressed in caudal progenitors and undetermined cell type (PAX3, GBX2, EN2), and in caudal neuronal types (TFAP2A, POU4F1).

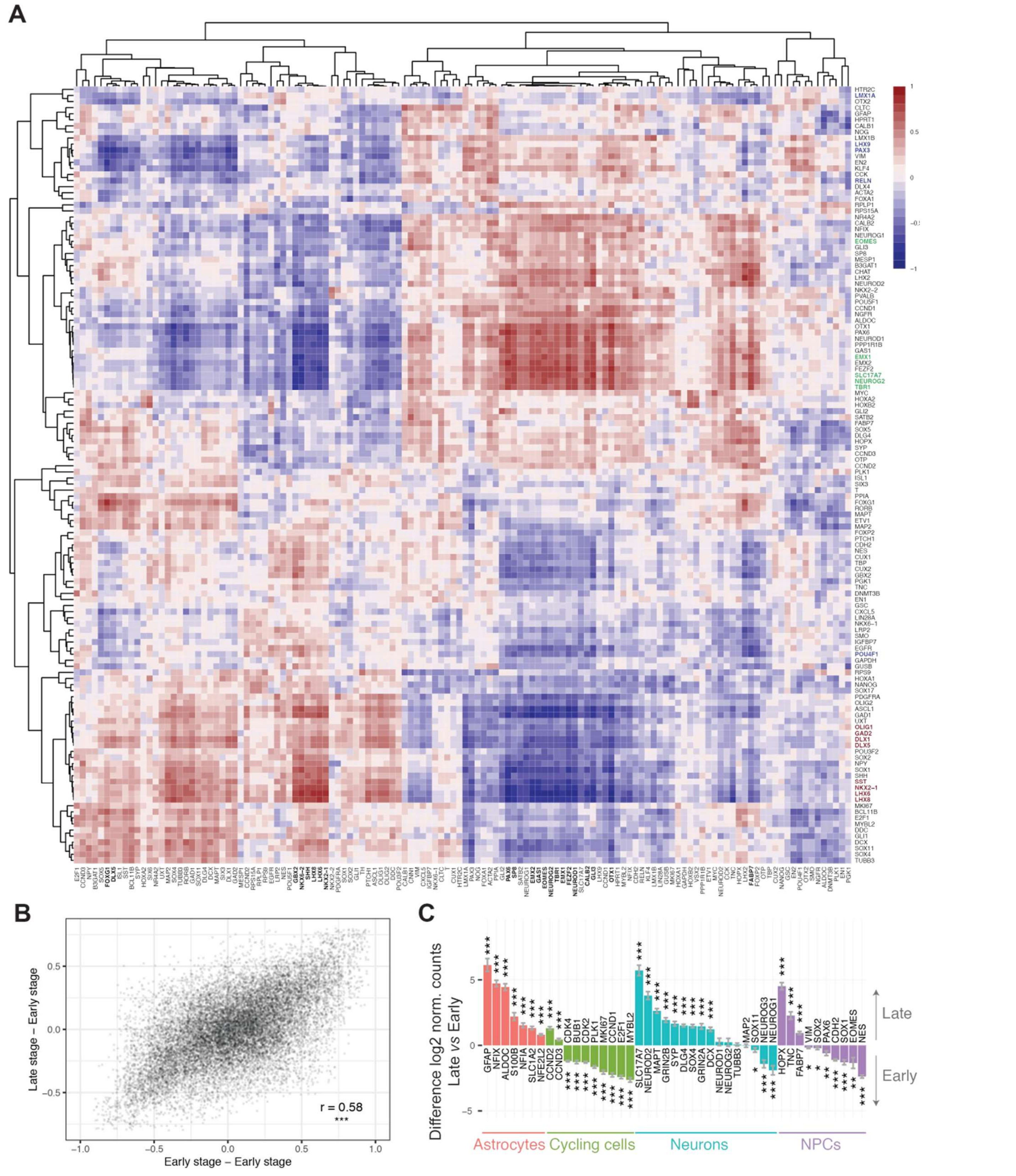

Figure S4. Late-stage expression reflects developmental temporal progression. Related to Figure 4.

(A) Pearson correlation between gene expression in late-stage differentiations (y-axis) and gene expression in early-stage differentiations (x-axis) for 44 time pairs. Late-stage cluster markers are reported in bold on the y-axis and colour coded

in green (dorsalised - LC2), red (ventralised - LC1), and blue (partially caudalised – LC3). Early-stage genes with an absolute correlation coefficient greater than 0.75 with any of these are indicated in bold on the x-axis, and selectively shown in Figure 4A.

(B) Relationship between expression correlation of any gene pair in the early-stage dataset and between the early-stage and late-stage dataset. The significant correlation between these two datasets indicates that expression of gene modules present at the early-stage is maintained at the late-stage, and therefore early-stage expression of these modules can predict late-stage expression.

(C) Average difference in expression between late-stage (~ 82 days post induction, dpi) and early-stage (~35 dpi) dorsalised differentiations. The expression of most astrocytic and neuronal genes increases, while most NPC and proliferation genes decrease over time (one-sample t-test on difference in paired late *vs* early samples, n = 30 pairs, mu = 0; FDR corrected p-values: <0.05 (*), <0.01 (**), <0.001 (***)). Error bars represent standard error.

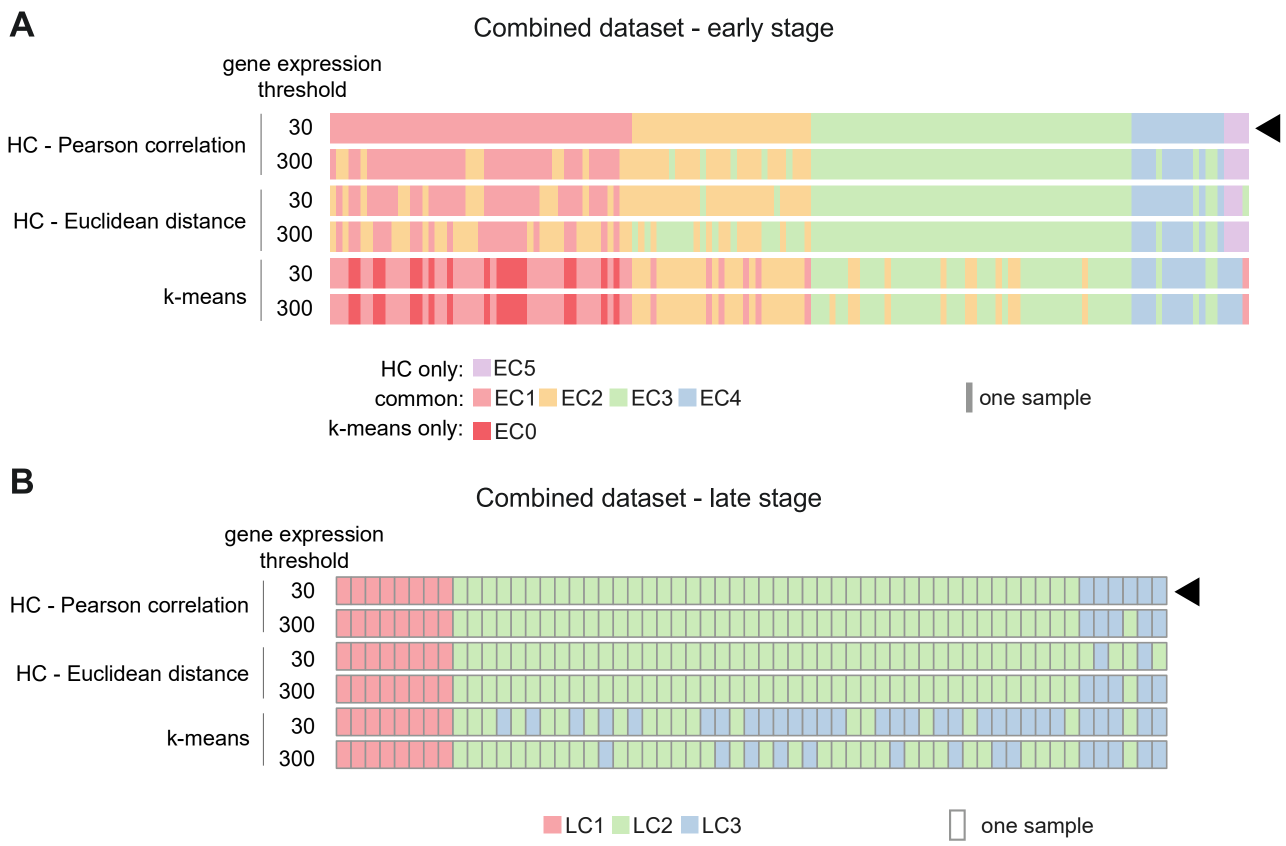

Figure S5. **Cluster similarity with different clustering approaches.** Related to Figure 4.

Separate rows represent cluster assignments for the combined early-stage dataset (A, 149 samples) or late-stage dataset (B, 57 samples) using indicated clustering method (k-means or HC: hierarchical clustering), clustering distance (Pearson correlation or Euclidean distance), and minimum threshold in at least one sample (30 or 300 normalised counts). Arrowheads indicate clustering used in the main text.

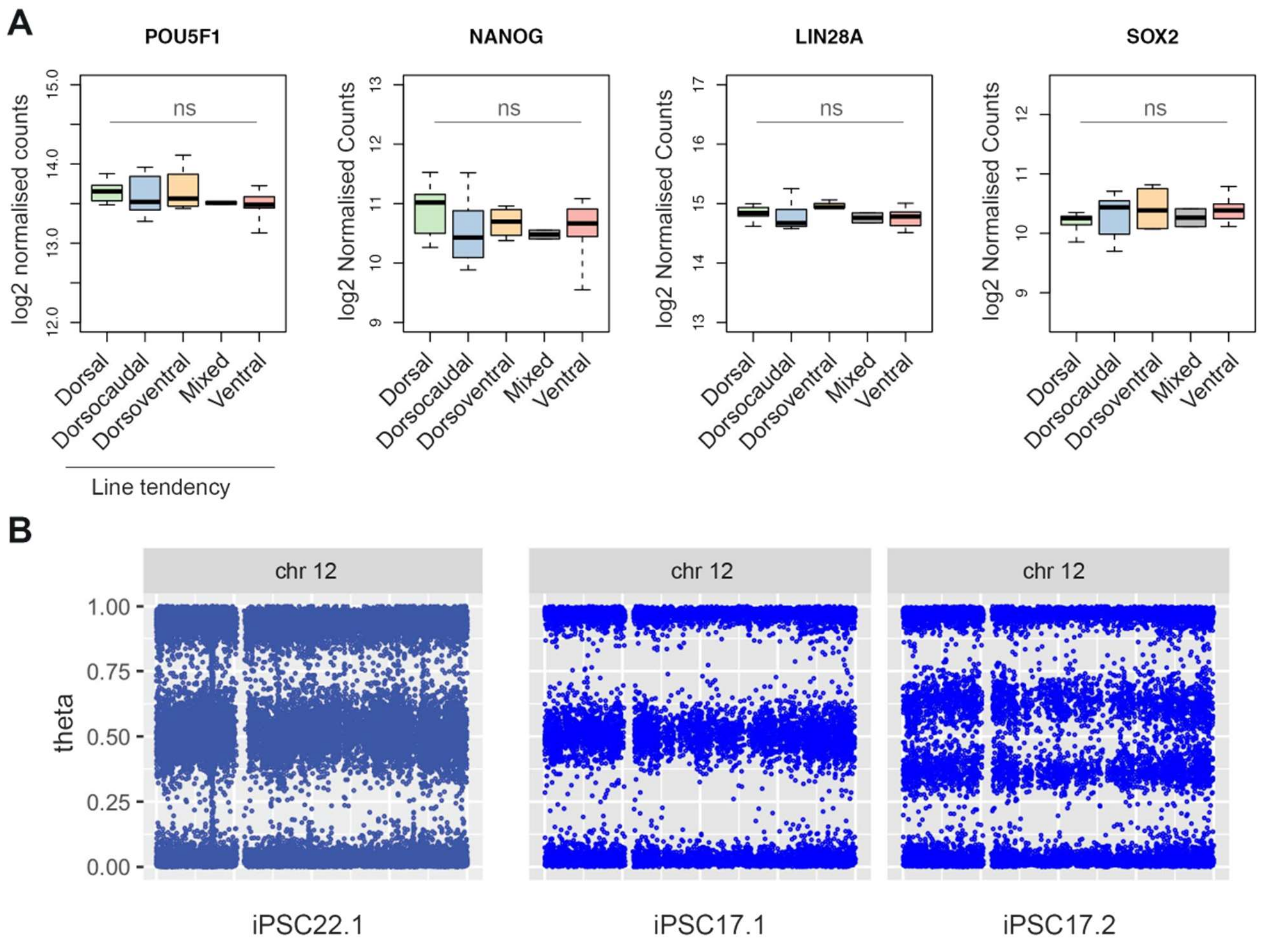

Figure S6. **Cell line contributions to differentiation outcome.** Related to Figure 5.

(A) Expression levels of key pluripotency genes grouped by patterning-proneness of the corresponding PSC line (one-way ANOVA, not significant). PSC lines per group: dorsal (7), dorsocaudal (5), dorsoventral (4), mixed (2), ventral (8).

(B) Allelic SNP ratio (theta) for single nucleotide polymorphism loci on chromosome 12 for a control line (iPSC22.1) and two clones derived from the same individual carrying a MAPT Ex10+16 mutation (iPSC17.1-2). The horizontal axis represents normalised chromosomal distance. The presence of 4 bands in iPSC17.2 indicates trisomy of chromosome 12.

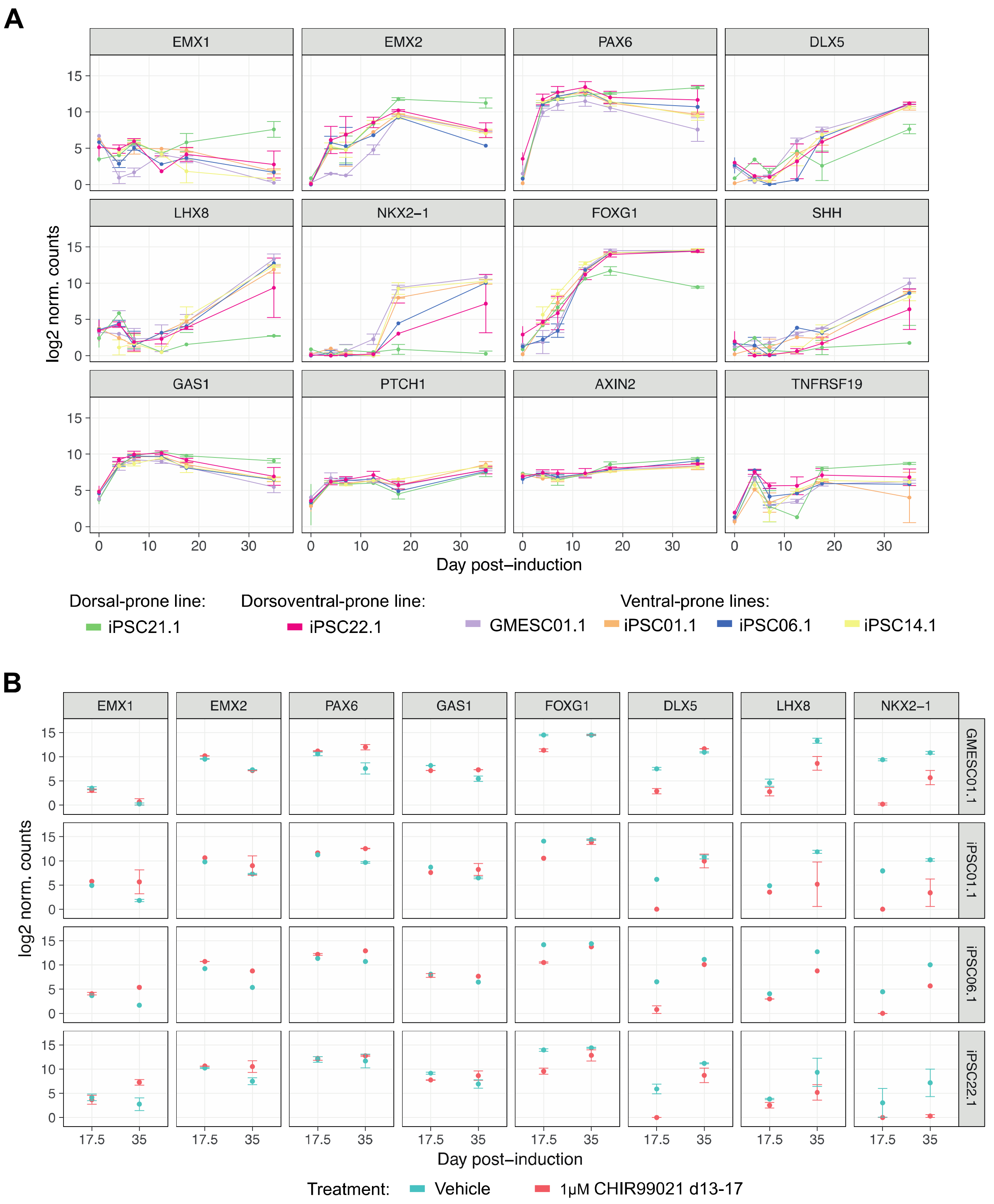

Figure S7. CHIR99021 treatment dorsalises gene expression in spontaneously-ventralising differentiations. Related to Figures 6 and 7.

(A) Gene expression timecourse during differentiation for selected genes associated with forebrain regions and Hedgehog or Wnt signalling pathways. Profiles from six PSC lines are shown, each profile represents the average of 1-3 differentiations. Notice the graded differences between a dorsal-prone line (iPSC21.1), a dorsoventral-prone line

(iPSC22.1), and four ventral-prone lines (GMESC01.1, iPSC01.1, iPSC06.1, iPSC14.1). Profiles represent average gene expression per line and error bars represent standard deviation.

(B) Log2 normalised count data corresponding to Figure 7F. Expression of key markers at ~17 and ~35 dpi is shown for four dorsoventral/ventral-prone lines treated with either vehicle or Wnt signalling activator CHIR99021 (1 μM) between 13-17 dpi. Number of differentiations (n = 2) except for iPSC01.1 at 17dpi and vehicle treated iPSC06.1, for which a single data point is shown. Error bars represent standard error.

SUPPLEMENTARY TABLES (see separate files)

Table S1. Nanostring Codesets Probe Sequences, Related to Figure 1

Table S2. Details of PSC Lines Used and their Differentiation Trends, Related to Figure 5

Table S3. Normalised Gene Expression Matrices for Assessment of Variation, Related to Figures 1-6

Table S4. Normalised Gene Expression Matrices for Temporal Dynamics and Effects of Signalling Manipulation, Related to Figures 6-7
