# Supplementary material for: Variable Outcomes in Neural Differentiation of Human PSCs Arise from Intrinsic Differences in Developmental Signaling Pathways
Source: Cell Rep. 2020 Jun 9;31(10):107732. doi: 10.1016/j.celrep.2020.107732 (PMC7296348; doi:10.1016/j.celrep.2020.107732)
Supplement: Document S2. Article plus Supplemental Information [file mmc6.pdf]

Cell Reports

Article

Variable Outcomes in Neural Differentiation of Human PSCs Arise from Intrinsic Differences in Developmental Signaling Pathways

Graphical Abstract

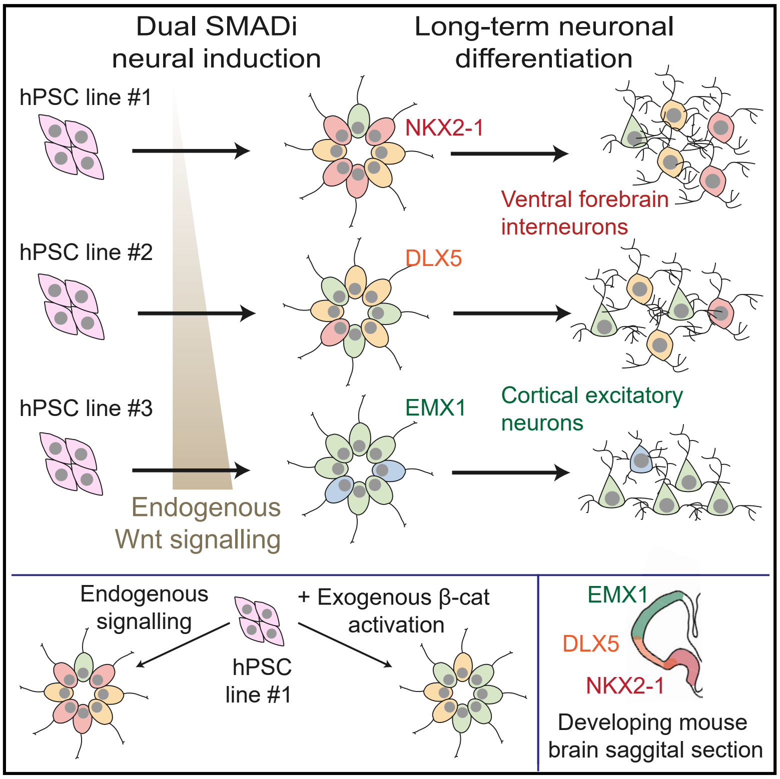

Highlights

- Analysis of 162 iPSC neural differentiation quantifies sources of variability
- Variation in outcomes occurs along developmental brain spatial and regional axes
- Variation depends on iPSC-line-specific differences in Wnt/ β-catenin signaling
- Effects of signaling differences can be rescued by exogenous pathway activation

Authors

Alessio Strano, Eleanor Tuck,
Victoria E. Stubbs, Frederick J. Livesey

Correspondence

r.livesey@ucl.ac.uk

In Brief

Long-term directed differentiation of human pluripotent stem cells (PSCs) can yield variable outcomes. Strano et al. assess variation in neural differentiation among PSC lines, revealing that variation occurs along developmental spatial and regional axes and depends on PSC-line-specific differences in endogenous Wnt/ β-catenin signaling, which can be rescued exogenously.

Strano et al., 2020, Cell Reports *31*, 107732
June 9, 2020 © 2020 The Authors.
https://doi.org/10.1016/j.celrep.2020.107732

CellPress

Cell Reports

CellPress
OPEN ACCESS

Article

Variable Outcomes in Neural Differentiation of Human PSCs Arise from Intrinsic Differences in Developmental Signaling Pathways

Alessio Strano,[1,2] Eleanor Tuck,[2] Victoria E. Stubbs,[1] and Frederick J. Livesey[2,3,*]
[1]The Wellcome Trust/Cancer Research UK Gurdon Institute & Department of Biochemistry, University of Cambridge, Cambridge, UK
[2]UCL Great Ormond Street Institute of Child Health, Zayed Centre for Research into Rare Disease in Children, University College London, 20 Guilford Street, London WC1N 1DZ, UK
[3]Lead Contact
*Correspondence: r.livesey@ucl.ac.uk
https://doi.org/10.1016/j.celrep.2020.107732

SUMMARY

Directed differentiation of human pluripotent stem cells varies in specificity and efficiency. Stochastic, genetic, intracellular, and environmental factors affect maintenance of pluripotency and differentiation into early embryonic lineages. However, factors affecting variation in *in vitro* differentiation to defined cell types are not well understood. To address this, we focused on a well-established differentiation process to cerebral cortex neural progenitor cells and their neuronal progeny from human pluripotent stem cells. Analysis of 162 differentiation outcomes of 61 stem cell lines derived from 37 individuals showed that most variation occurs along gene expression axes reflecting dorsoventral and rostrocaudal spatial expression during *in vivo* brain development. Line-independent and line-dependent variations occur, with the latter driven largely by differences in endogenous Wnt signaling activity. Tuning Wnt signaling during a specific phase early in the differentiation process reduces variability, demonstrating that cell-line/genome-specific differentiation outcome biases can be corrected by controlling extracellular signaling.

INTRODUCTION

The discovery of human somatic cell reprogramming to generate pluripotent stem cells (PSCs) and the development of methods to differentiate PSCs to a range of cell fates have provided unprecedented access to human cell types of interest for a variety of purposes (Clevers, 2016; Shi et al., 2017; Simunovic and Brivanlou, 2017). Expansion in the scope of PSC differentiation approaches and in the number of generated PSC lines has highlighted a significant degree of variation in developmental outcomes in these experimental systems. First, spatial effects affecting cell-cell interactions and heterogeneity in signaling and transcription can lead to variation in developmental fate between PSCs differentiated together (Bauwens et al., 2008; Chambers et al., 2009; Kobayashi et al., 2009; Moya et al., 2014). Second, PSC lines vary in their propensity to differentiate into particular embryonic lineages (Hu et al., 2010; Kajiwara et al., 2012; Osafune et al., 2008; Siller et al., 2016), with epigenetic memory of cell of origin (Bar-Nur et al., 2011; Kim et al., 2010), endogenous signaling pathway activation (Kattman et al., 2011; Nazareth et al., 2013; Nostro et al., 2011), and genetic variability (Carcamo-Orive et al., 2017; DeBoever et al., 2017; Kilpinen et al., 2017), all implicated as contributing factors. Finally, when PSCs are used to derive multipotent progenitor cells, any variation in the proportions of distinct cell types they generate can provide an additional confound in cross-differentiation comparisons (Volpato et al., 2018). To date, studies of the underlying causes of PSC variation have largely focused on maintenance of pluripotency and differentiation into early embryonic lineages; conversely, the causes of variation during directed differentiation of PSCs into various somatic cell types and tissues are generally not as well understood.

Stem cell models of the human brain and cerebral cortex are of particular interest, given that they provide relevant and otherwise largely inaccessible cell types to enable the investigation of a broad range of human biology, including neurodevelopmental and neurodegenerative disorders (Moore et al., 2015; Shi et al., 2012a), the action of infective pathogens such as Zika virus (Dang et al., 2016; Qian et al., 2016; Zhou et al., 2017), and the evolution of the primate cerebral cortex (Mora-Bermúdez et al., 2016; Otani et al., 2016). Several methods have been published to generate dorsal forebrain or cerebral cortex in adherent and organoid culture, with differences in signaling pathway manipulation, brain regions generated, and suitability for different applications and research questions (reviewed in Kelava and Lancaster, 2016).

To investigate the sources of variation in developmental fate and culture composition during directed differentiation of PSCs, we focused on one cortical differentiation method (Shi et al., 2012b), the core of which is the widely used dual-SMAD inhibition (Chambers et al., 2009). To understand the degree and nature of variation in developmental outcomes during neural

This is an open access article under the CC BY license (http://creativecommons.org/licenses/by/4.0/).

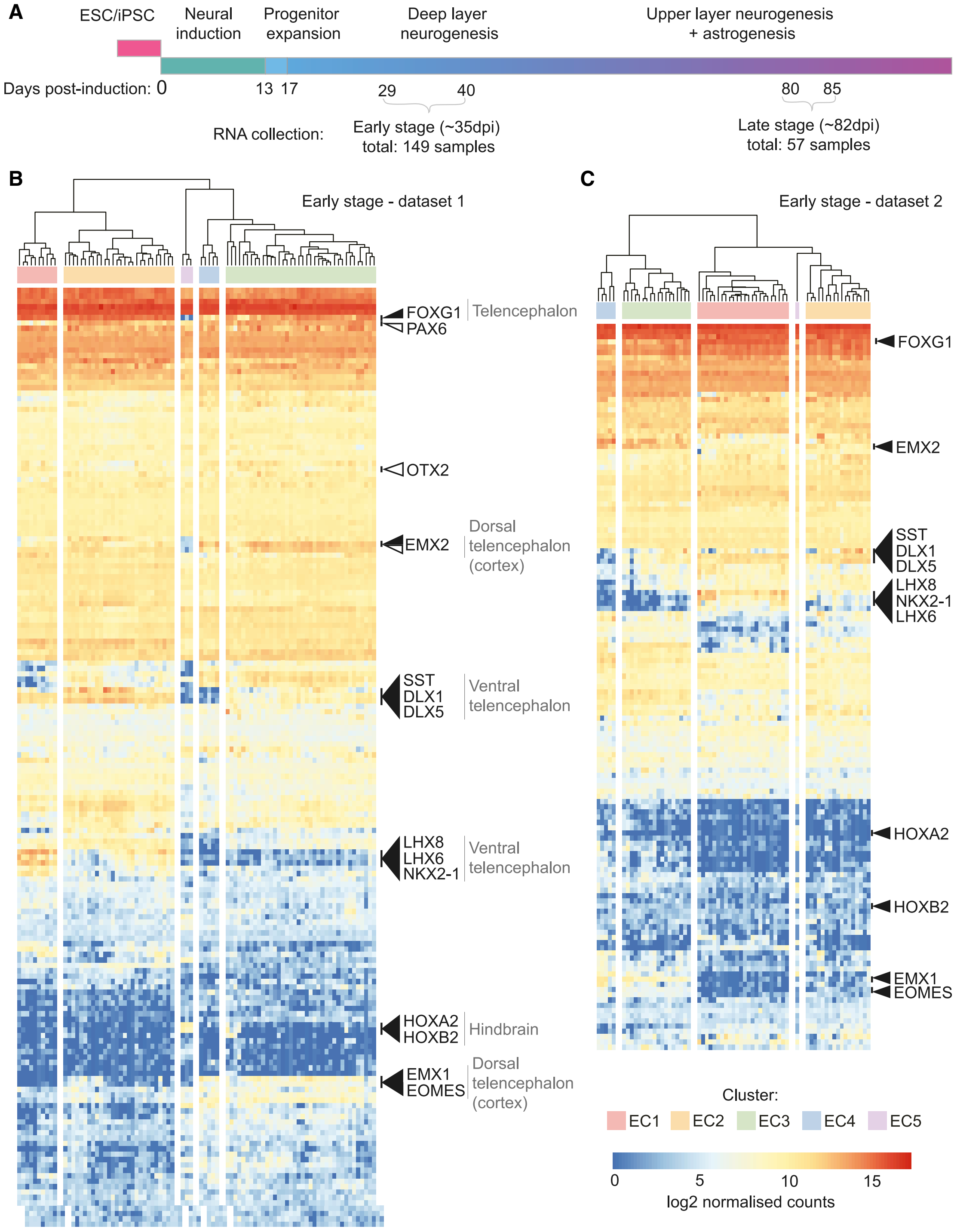

Figure 1. Gene Expression Profiling in 84 Directed Differentiations Highlights Broad Transcriptional Similarity and Specific Differences in Expression of Regional Brain Genes

(A) Protocol used to differentiate cortical cultures from PSCs. The early and late stages analyzed are highlighted.

(B) Hierarchical clustering of gene expression from 84 early-stage differentiations profiled with Codeset1. Clusters are named early cluster 1 (EC1)–EC5. Highly expressed cortical development genes are indicated with white arrowheads. Variation was observed in expression of transcripts specific to the telencephalon

(legend continued on next page)

directed differentiation of PSCs, we analyzed both regional identity and neural cell types generated in a large dataset of 162 cortical differentiations from 61 PSC lines. We find that most variation occurs specifically along spatial gene expression axes known from *in vivo* brain development, with a clear line-dependent bias. Regional drift from dorsal forebrain/cortex, the target tissue, occurs, at least in part, due to differences in endogenous signaling pathway activation, most notably of Wnt signaling. Manipulation of this pathway to channel signaling within a defined time window corrects for those biases, indicating that such biases are not insurmountable and that applying developmental biology principles to channel-directed differentiation enables more precise engineering of outcomes.

RESULTS

Analysis of a Large Number of Directed Differentiations Highlights Overall Reproducibility, with Some Variation in Spatial Identities

To study variation between directed differentiations of PSCs into cortical tissue, we focused on a previously characterized and well-established method for 2D cortical differentiation based on dual-SMAD inhibition and retinoic acid signaling, with otherwise minimal signaling manipulation (Figure 1A) (Shi et al., 2012b, 2012c). This directed differentiation approach generates PAX6^{+} OTX1/2^{+} dorsal forebrain neural progenitor cells that recapitulate *in vivo* cerebral cortex lineage progression, dividing and differentiating over 2–3 months to produce deep layer neurons, upper layer neurons, and astrocytes in a temporal order akin to that observed during *in vivo* development (Shi et al., 2012c).

To investigate in-depth variation in differentiation outcomes, we measured gene expression using the Nanostring nCounter platform, which enabled us to compare differentiations performed over several months (Figures S1A and S1B). We profiled 162 directed differentiations at two time windows in the differentiation process (Figure 1A, Table S3), analyzing a total of 206 RNA samples. The two stages analyzed capture an early stage of neural progenitor proliferation and deep layer neurogenesis (29–40 days post-differentiation; dpi), and a late stage of upper layer neurogenesis and gliogenesis (80–85 dpi) (Figure 1A) (Shi et al., 2012c).

We focused our analyses on the expression of a curated panel of genes indicative of cell or spatial identity in the developing embryo based on developmental and stem cell biology (Evseenko et al., 2010; Flames et al., 2007; Maroof et al., 2013; Menendez et al., 2013; Merkle et al., 2015; Molyneaux et al., 2007; Mormone et al., 2014; Najm et al., 2011; Nicholas et al., 2013; Shaltouki et al., 2013; Teo et al., 2012; Tsankov et al., 2015; Whitfield et al., 2006; Yasunaga et al., 2005), previous gene expression studies of similar differentiations (Floruta et al., 2017; Yao et al., 2017), and recurrent drivers of variation in our own unpublished RNA sequencing (RNA-seq) datasets. This panel included genes specifically expressed in particular cell types, germ layers, and developing brain regions, as well as genes associated with different cell states (e.g., cell cycle and apoptosis) or involved in key developmental signaling pathways, including Hedgehog, Notch, Wnt, and Fgf components, for a total of 200 (Codeset 1) and 156 (Codeset 2) gene probes (Table S1).

First, we analyzed variation in cell composition in an early-stage dataset comprising 84 separate differentiations of 35 different cell lines generated from 25 individuals. All directed differentiations at this stage had low to no expression of genes associated with pluripotency or mesodermal and endodermal fates (Figures S1C and S1D), confirming efficient neurectoderm differentiation and demonstrating that differences in early germ layer choice are not major contributors to variation at this stage. Hierarchical clustering of normalized gene counts revealed a similar gene expression profile across differentiations (Figure 1B), with typically high expression of genes expressed during early cortical development (PAX6, EMX2, and OTX2; Figure 1B, white arrowheads). However, genes with variable expression among directed differentiations included genes expressed in the cerebral cortex (EMX1/2 and EOMES), the ventral forebrain (NKX2-1, LHX6/8, and DLX1/5), and the hindbrain (HOXA2 and HOXB2) (Figure 1B, black arrowheads), indicating variable proportions of non-cortical cells within some of the directed differentiations.

Analysis of a second, independent dataset of another 65 early-stage differentiations of 28 lines (8 lines present in dataset 1 plus 20 additional lines) from 22 individuals (Figure 1C) confirmed the observation of a variable proportion of non-cortical cells in a subset of differentiations. Both datasets could be divided in 5 clusters with similar expression profiles, which we defined as early clusters 1–5 (EC1–EC5) to distinguish them from clusters at later stages of differentiation (discussed later). Immunostaining for cell-type-specific antigens (TBR1, CTIP2, GABA, TBR2, and SOX2) in 55-dpi differentiations, we quantified the fractional composition of neuronal and neural progenitor types and found it to be significantly correlated with gene expression at the same age (Figure S1E), confirming that variation in gene expression is representative of variation in cell composition.

Variation in Gene Expression among Early-Stage Cortical Differentiations Corresponds to *In Vivo* Regional Gene Expression

Principal-component analysis (PCA) was applied to identify genes that contributed most to the observed variation within early-stage gene expression (Figure 2A). Most differentiations (81/84, 96%) were part of a large group spread along principal component 1 (PC1), while three differentiations separated on PC2 (Figure 2A). The genes with the highest loadings for PC1 were genes that are differentially expressed along the dorsoventral axis in the developing forebrain *in vivo*, including genes

(FOXG1), the ventral telencephalon (LHX8, LHX6, NKX2-1, DLX1, and DLX5), the hindbrain (HOXA2 and HOXB2), and the dorsal telencephalon (cortex) (EMX1, EMX2, and EOMES), indicated with black arrowheads.

(C) Replicating the patterns observed in (B), genes associated with specific brain regions are highly variable across differentiations in a second independent dataset of 65 early-stage differentiations profiled with Codeset2.

See also Figure S1.

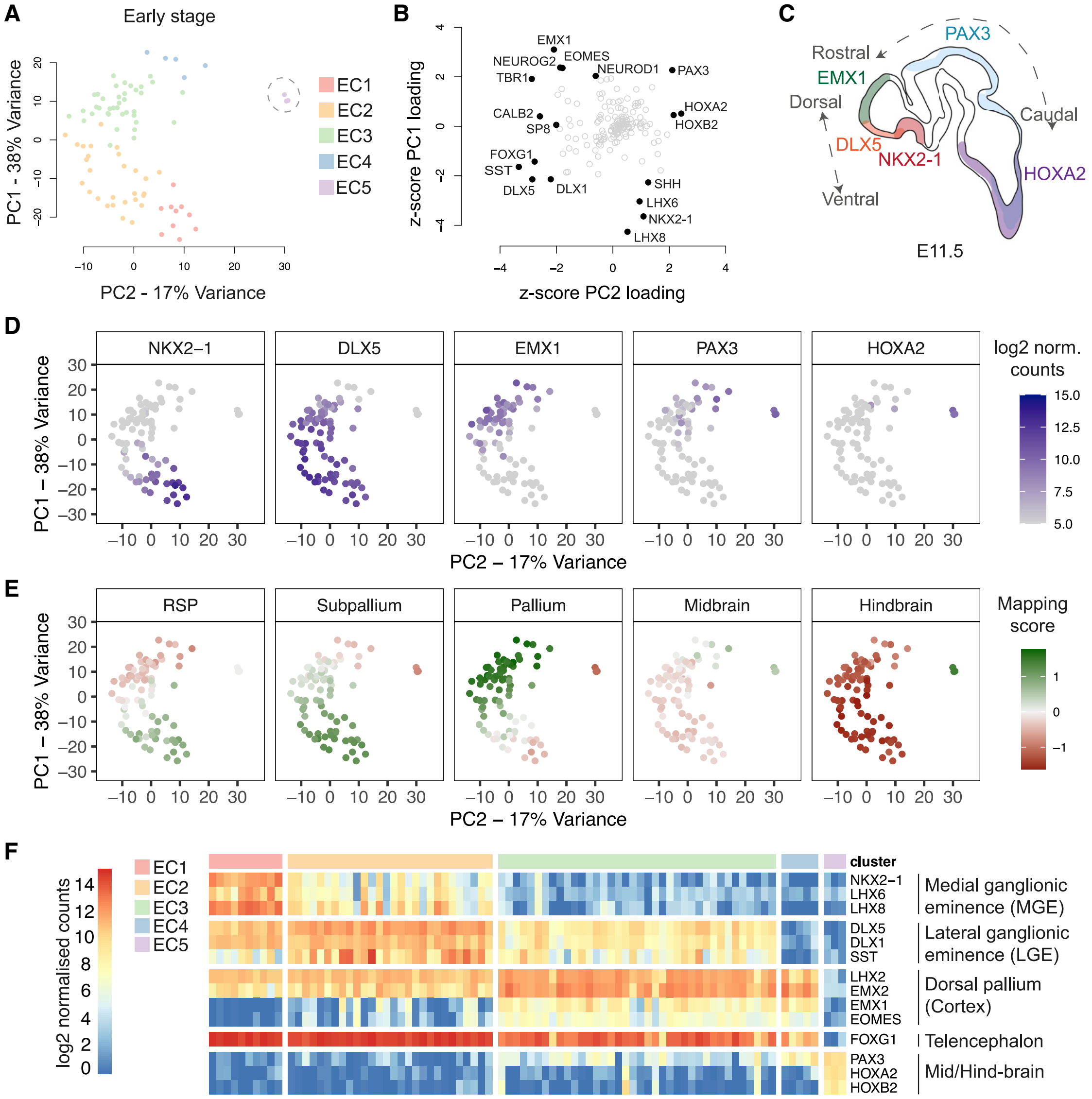

Figure 2. Analysis of Variation in Gene Expression at Early Stage Reveals Differences in Spatial Identity Reflecting *In Vivo* Dorsoventral and Rostrocaudal Axes

(A) Principal-component analysis (PCA) of early-stage gene expression data (84 samples, 174 genes); samples are plotted along the two components explaining most gene expression variation and colored by cluster. Caudal outliers are circled. EC, early cluster.

(B) Gene contributions to variation in the early-stage dataset plotted using the *Z*-scored loadings for PC1 and PC2 as coordinates. Highest contributors (absolute *Z* scores >2) are labeled.

(C) The distribution of highest loading genes in (B) is correlated with the gene expression patterns along the dorsoventral and rostrocaudal axes *in vivo*.

(D) Expression of selected high-loading genes along PC1–PC2.

(E) Mapping to 5 regions of the E11.5 Allen Developing Mouse Brain Atlas based on correlated expression of variable genes. RSP, rostral secondary prosencephalon.

(F) Expression heatmap of selected high-loading genes in individual differentiations.

See also Figure S2.

expressed specifically in the cortex/dorsal pallium (EMX1, NEUROG2, and EOMES) and medial ganglionic eminence (MGE) (LHX8, NKX2-1, and LHX6).

In contrast, the highest loading genes for PC2 were genes whose expression is restricted to the midbrain and/or hindbrain (HOXA2, HOXB2, and PAX3) or the forebrain (SST, DLX5, TBR1, and FOXG1) (Figure 2B). Additionally, samples positioned toward the middle of PC1 displayed notably high expression of DLX1 and DLX5 (Figures 2B–2D), two genes expressed in the MGE and in the lateral and caudal ganglionic eminences (LGE and CGE, respectively) *in vivo*. Given the lack of correlation with expression of MGE-defining NKX2-1 and LHX6/8, we

interpret the DLX expression as indicating cells of LGE and possibly CGE fate (Flames et al., 2007; Sussel et al., 1999). The overall distribution of gene expression along PC1 and PC2, therefore, approximately corresponds to dorsoventral and rostrocaudal axes of gene expression in the developing brain *in vivo* (Figures 2B–2D and S2A). Consistent with this, mapping of individual differentiations to five regions of the embryonic day 11.5 (E11.5) mouse brain from the Allen Developing Mouse Brain Atlas produced a gradient of mapping to ventro-rostral, dorso-rostral, and dorso-caudal brain regions along PC1 and PC2 (Figure 2E).

These results and differential gene expression across early-stage clusters (Figure S2B) indicate variable contributions to each differentiation of cell types from cortex, LGE, MGE, and mid-hindbrain (Figure 2F). Furthermore, expression of genes enriched in the ventral forebrain was highly positively correlated with expression of neuronal differentiation genes (MAPT, DLG4, SYP, DCX, MAP2, SOX4, SOX11, and TUBB3) (Figure S2C), suggesting that differences in patterning (dorsal versus ventral forebrain) may also be associated with differences in the proportion of progenitors and neurons, with increased terminal neuronal differentiation occurring in more ventralized cultures.

Late-Stage Gene Expression Varies along Developmental Spatial Axes

To analyze variation in outcomes of directed differentiations and the neuronal types generated after long-term culture, we studied 44 differentiations from 33 lines for which we collected RNA at 80–85 dpi. At this stage, human-directed differentiations typically contain both early-born, deep layer neuronal types and late-born, upper layer neuronal types, as well as astrocytes and late-stage neural progenitor cells (Otani et al., 2016; Shi et al., 2012c; van de Leemput et al., 2014). Applying PCA and hierarchical clustering to the expression data, we found dorsoventral identity to also be the main source of variation among differentiations at this stage (Figures 3A–3F). Among genes that had highest loading for PC1 were two genes specific to either excitatory or inhibitory neurons (the glutamate transporter SLC17A7 and the GABA synthesizing enzyme GAD2; Figure 3B), which were anti-correlated. This anti-correlation demonstrated that variation in dorsoventral spatial identity was accompanied by differences in the proportions of each neuronal type, consistent with their differential origin along the forebrain dorsoventral axis *in vivo*.

The majority of differentiations we assessed at this late stage were cortical with some LGE contributions (36/44, late cluster LC2) and best matched to the pallium when mapped to E15.5 samples in the Allen Developing Mouse Brain Atlas (Figure 3E). Three differentiations included a clear, MGE-like gene expression pattern (late cluster LC1), and 5 differentiations (late cluster LC3) that separated from the rest along PC2 (Figure 3A) were characterized by lower expression of the forebrain-specific gene FOXG1 and higher expression of PAX3, RELN, TFAP2A, LHX9, POU4F1, GBX2, and EN2 (Figures 3F and S3A). With the exception of RELN and LHX9, these genes are classifiers of midbrain regions and potentially also neural-crest-derived lineages that arise from regions spanning from the diencephalon to the hindbrain (Santagati and Rijli, 2003). Analysis of the expression pattern of these genes through *in situ* hybridization data from the Allen Developing Mouse Brain Atlas (Figure S3B) and from a single-cell sequencing study of human PSC-derived forebrain differentiations (Yao et al., 2017) (Figure S3C) confirmed this hypothesis. Consistent with our interpretation, most LC3-associated genes were expressed exclusively or at higher levels in diencephalic/midbrain regions in mouse at E15.5 and were expressed at higher levels in the midbrain-lineage populations in the single-cell sequencing data (Figure S3C). Based on these observations, we classified the late-stage differentiations as dorsalized (LC2), partially caudalized (LC3), and highly ventralized (LC1). Moreover, we note that 6/36 dorsalized differentiations also included expression of OTP and SIM1 (Figure 3F), two genes highly expressed in the hypothalamus, suggesting the occasional contributions of hypothalamic cell types to the differentiations.

Early Differences in Regional Gene Expression Are Predictive of Late-Stage Fates

To explore whether early gene expression is predictive of late-stage differentiation outcomes, we merged datasets obtained with the two versions of our codeset and calculated Pearson correlation coefficients between expression of genes associated with late-stage clusters and gene expression at the early stage (44 pairs). Consistent with regional identity being established early during the differentiation process, the highest correlations for late-stage expression of cortex-associated genes were with expression of cortex-associated genes at the early stage, and similarly for ventral forebrain genes (Figures 4A and S4A). Stronger correlations for late-stage expression of caudal brain genes were negative correlations with forebrain-expressed genes FOXG1 and DLX5 (Figure 4A), indicating that caudalization of differentiations can be predicted based on low expression of forebrain markers. Correlation coefficients between expression of any pairs of genes at the late and early stages in an individual differentiation were correlated to correlation coefficients between those genes within the early-stage dataset, indicating the presence of gene modules which are co-expressed over time (Figure S4B). Direct comparison of cortical differentiations profiled at both the early and late stages highlighted significant gene expression changes consistent with progressive neurogenesis and astrogliogenesis over time (Figure S4C), as previously reported (Shi et al., 2012c; van de Leemput et al., 2014).

Comparing the clustering classification between the two temporal stages for 44 differentiations allowed us to assess whether the high correlation of gene expression patterns at the two stages was reflected in consistent cluster assignment. In this scenario, a perfectly matching cluster assignment at the two stages would indicate complete predictability of late-stage differentiation outcome based on early-stage data. Performing hierarchical clustering on these samples, we found that 7 of 9 differentiations that were classified as highly ventralized at the early stage were also classified as highly ventralized at the late stage and that 5 of 6 of differentiations classified as partially caudalized at the early stage were also classified as partially caudalized at the late stage (Figure 4B, k = 3). Early-stage, partially ventralized differentiations did not separate from dorsalized differentiations at the late stage, indicating that this category has minimal predictive value (Figure 4B, k = 4). Early-stage clustering classification

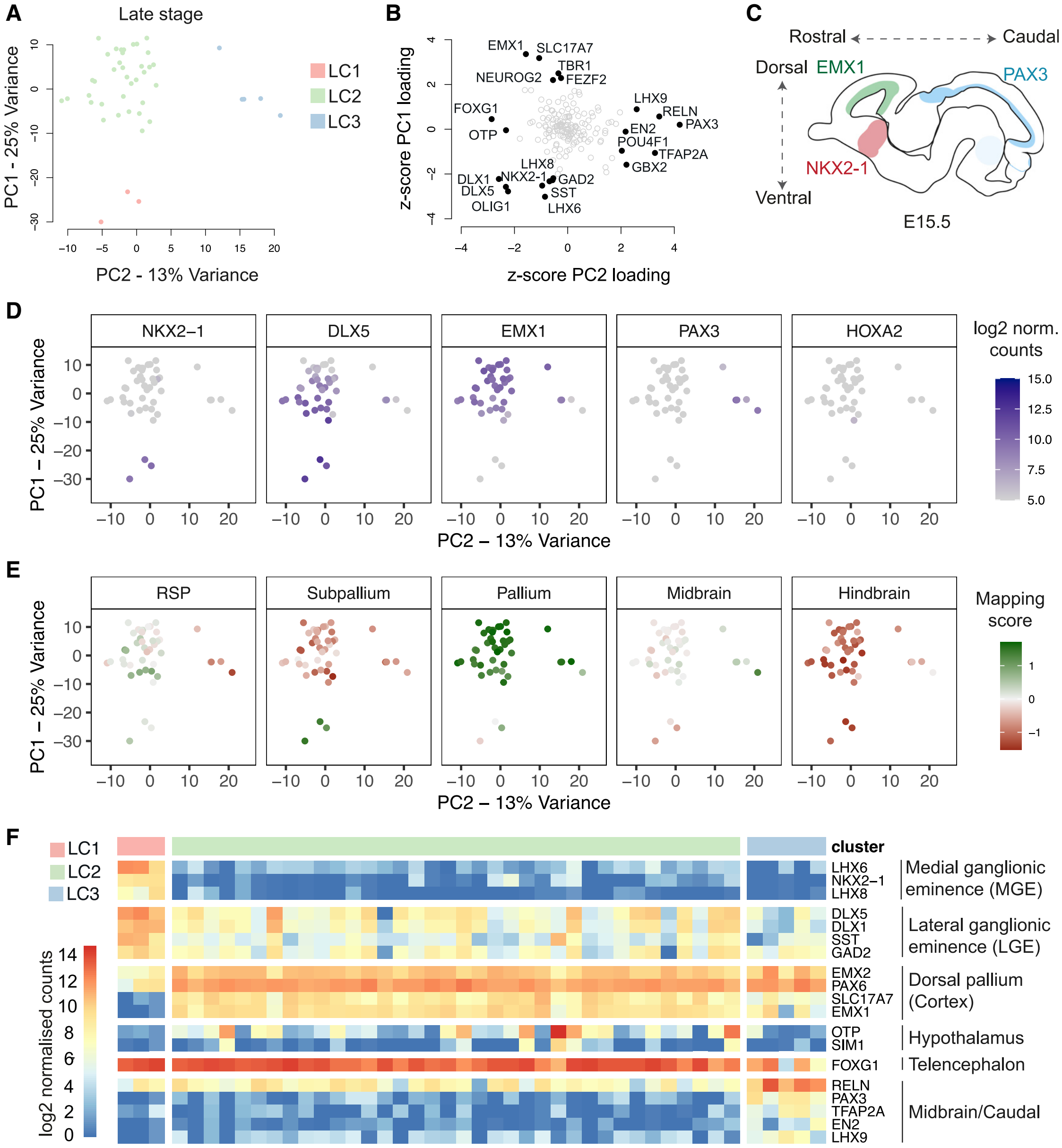

Figure 3. Developmental Patterning Axes Are the Main Drivers of Variation in Late-Stage Differentiations

(A) PCA of late-stage gene expression data (44 samples, 171 genes) with samples colored by cluster. LC, late cluster.

(B) Gene contributions to variation in the late-stage dataset plotted using the *Z*-scored loadings for PC1 and PC2 as coordinates. Highest contributors (absolute *Z* scores >2) are labeled.

(C) The distribution of highest loading genes in (B) is correlated with the gene expression patterns along the dorsoventral and rostrocaudal axes *in vivo* and resembles the first two principal components in the early-stage data.

(D) Expression of selected high-loading genes along PC1–PC2.

(E) Mapping to 5 regions of the E15.5 Allen Developing Mouse Brain Atlas based on correlated expression of variable genes. RSP, rostral secondary prosencephalon.

(F) Expression heatmap of selected high-loading genes in individual differentiations and interpretation summary showing variable contribution of different regional identities to composition of differentiations in three late-stage clusters.

See also Figure S3.

could, therefore, predict late-stage classification into the highly ventralized, dorsalized, and partially caudalized groups (k = 3) for 93% (41/44) of the differentiations (Figure 4B).

Although our cluster assignment was similar across different clustering methods, distance metrics, and thresholds tested (Figure S5), clustering approaches to classification rely on large

CellPress
OPEN ACCESS

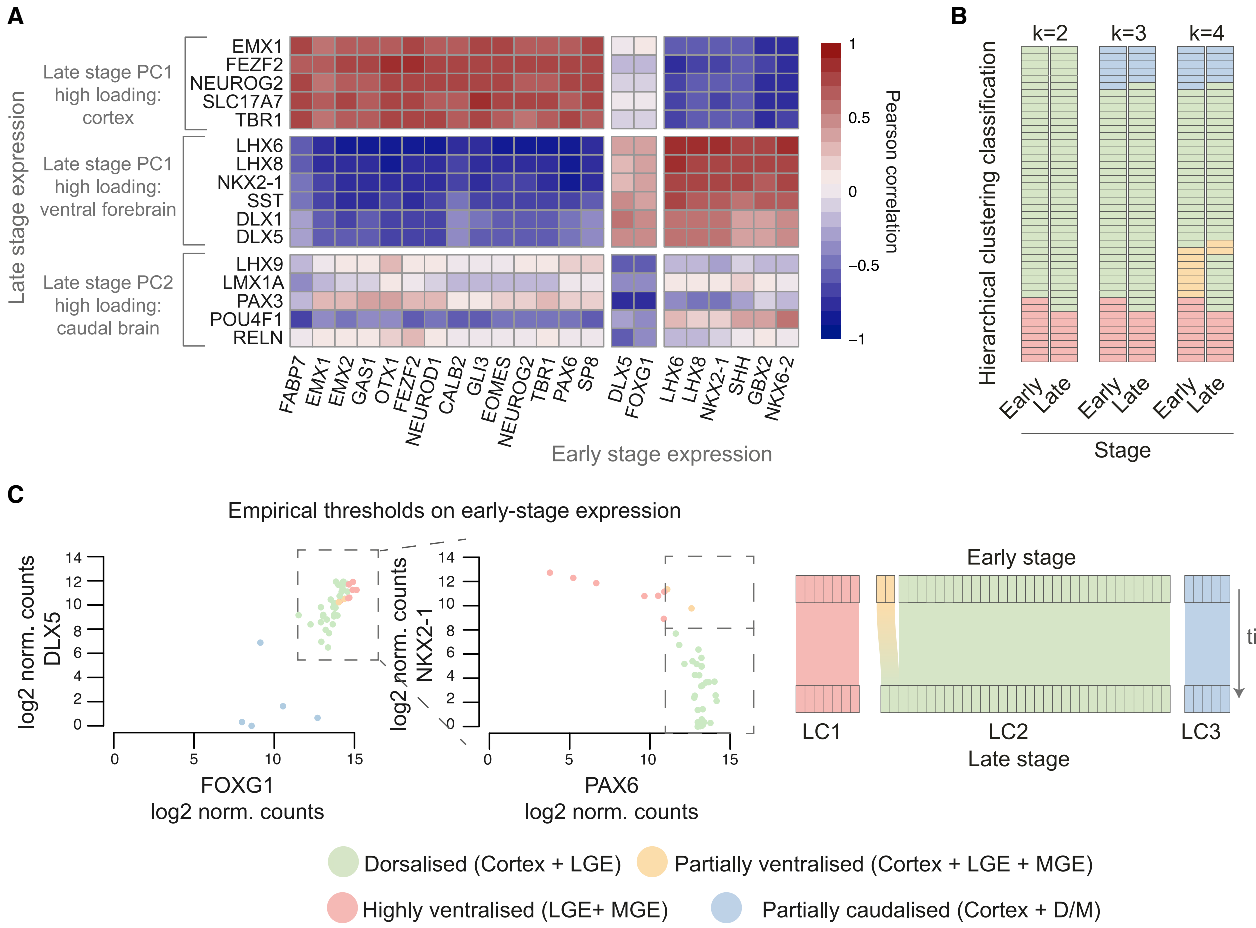

Figure 4. Early-Stage Gene Expression Is Predictive of Late-Stage Expression and Differentiation Outcomes

(A) Heatmap of Pearson correlation coefficients between expression of late-stage cluster markers and early-stage gene expression in 44 time pairs. Only early-stage genes with an absolute correlation coefficient greater than 0.75 for at least one of the late-stage markers are included.

(B) Correspondence between early-stage and late-stage cluster assignmentS mapped for 44 differentiations when two, three, or four clusters are considered.

(C) Late-stage cluster outcome was used to establish acceptable thresholds of expression of predictive genes at the early stage: low DLX5 and FOXG1 expression were used to classify differentiations as partially caudalized. For the remaining rostral differentiations, high NKX2-1 expression and low PAX6 expression were used to classify differentiations as highly ventralized, while samples with high PAX6 and high NKX2-1 expression were classified as partially ventralized. Correspondence of empirical classification at early-stage with late-stage cluster classification is mapped on the right.

See also Figures S4 and S5.

sample numbers and can be affected by which samples and genes are included. Therefore, we sought to establish quality control measures based on absolute thresholds of early-stage expression of a limited number of genes. This approach exploits the observed high correlation between markers of specific tissue types (Figure 4A) and should also be more translatable to lower throughput technologies. We focused on two pairs of genes (NKX2-1 and PAX6; and FOXG1 and DLX5) whose early-stage expression displayed high positive or negative correlation with expression of late-stage cluster markers (Figure 4A).

Using the 44 pairs of early- and late-stage samples, we determined thresholds of early-stage gene expression that separated dorsalized differentiations (LC2) from partially caudalized (LC3) and highly ventralized (LC1) ones, obtaining early-stage classifications that fully matched those of the late-stage differentiations (Figure 4C). This analysis highlighted an inverse relationship between NKX2-1 and PAX6 expression (Figure 4C): the differentiations with high expression of both genes, which are classified as partially ventralized by our thresholds, are those that the clustering approach classifies as highly ventral at the early stage but more cortical-like at the late stage. We speculate that this may result from more extensive proliferation of cortical progenitors compared to ventral progenitors, as suggested by the correlation noted earlier between genes expressed in the ventral forebrain and neuronal differentiation genes (Figure S2C).

Cell-Line-Intrinsic Contributions to Variation in Differentiation Outcomes

A key question is whether different lines have reproducible propensities to acquire cortical and non-cortical regional identities. To investigate this, we used the gene expression thresholds

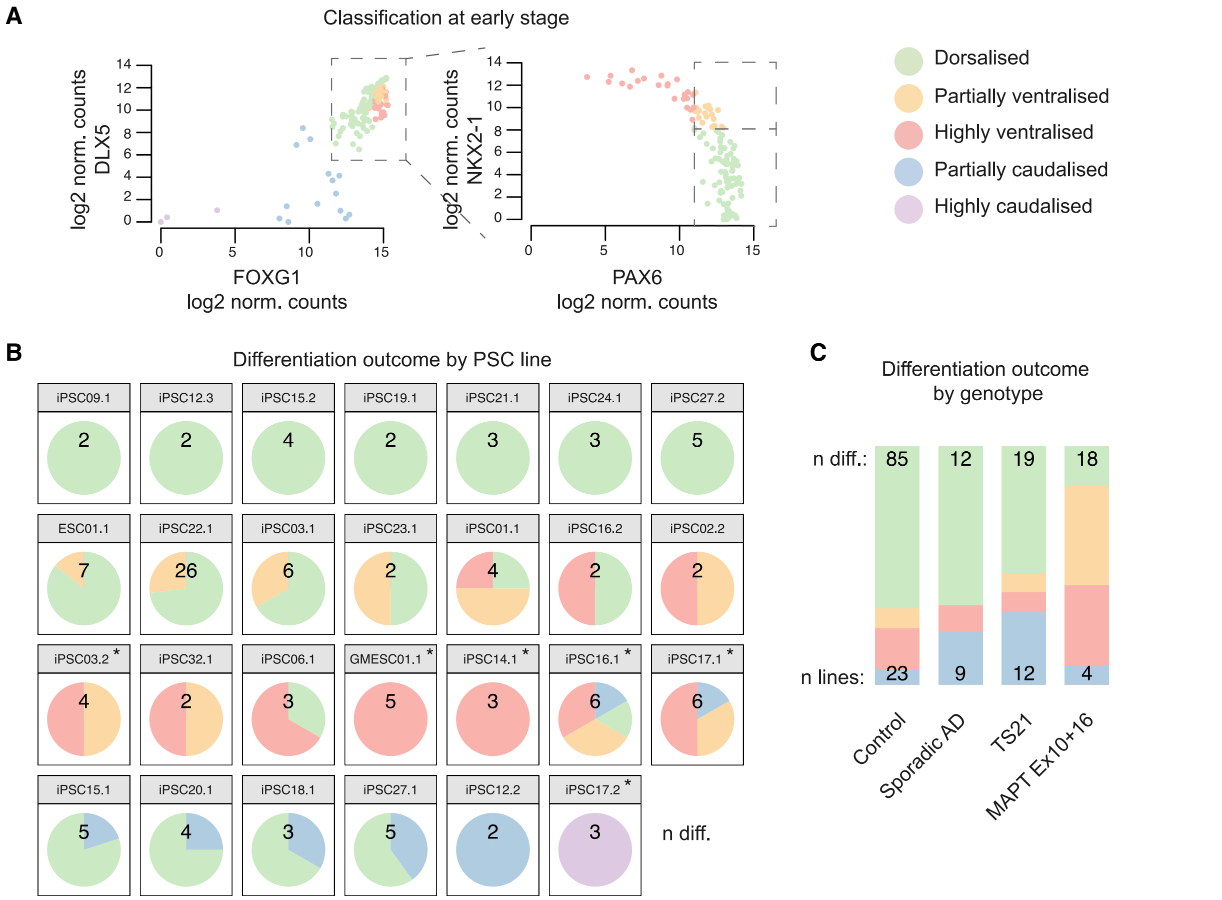

Figure 5. Cell-Line-Specific Variation in Spatial Identity of Neural Differentiations

(A) Classification of differentiations from early-stage data as dorsalized (green), partially ventralized (orange), highly ventralized (red), and partially caudalized (blue) using thresholds determined in Figure 4C. Additionally, samples with extremely low DLX5 and FOXG1 expression (belonging to EC5) were classified as highly caudalized (purple).

(B) Differentiation outcome frequency plotted by PSC line for lines with at least two differentiations. Names are formatted as individual.clone; numbers indicate separate differentiations per line. Asterisks indicate lines for which the Wilson 95% confidence interval of the difference in dorsal differentiation frequency compared to overall frequency does not include zero.

(C) Differentiation outcome frequency plotted by genotypes for which at least three cell lines were induced. MAPT Ex10+16, frontotemporal-dementia-causing exon 10 splicing mutation in gene encoding tau protein; TS21, trisomy 21 (Down syndrome); AD, Alzheimer's disease. Frequencies were normalized to account for the variable number of differentiations per line.

See also Figure S6.

previously determined (Figure 4C) to classify all 149 differentiations as dorsalized (86), partially ventralized (23), highly ventralized (23), partially caudalized (14), or highly caudalized (3) (Figure 5A). For further analysis, we focused on differentiations from PSC lines for which we had information on at least 2 differentiations.

The majority (16/27; 59%) of PSC lines we studied generated dorsalized differentiations most of the time, with occasional partial ventralization or caudalization (Figure 5B). Of the lines for which one or more differentiations were not dorsalized, 18/20 lines produced differentiations with either ventralized or caudalized gene expression, but not both. Thus, we observed line-specific differentiation tendencies affecting the acquisition of regional identity and the range of differentiation outcomes that are produced. Furthermore, although we did not investigate any operator-dependent effects, we note that none of the 26 differentiations of line iPSC22.1, performed by 7 different operators, were caudalized or highly ventralized, suggesting that differences between experienced operators are less important than differences between PSC lines (Figure 5B). Based on the type of non-dorsalized differentiations we observed, we classified PSC lines as either ventral or caudal prone (Table S2). Dorsal-, ventral-, or caudal-prone PSC lines had similar expressions of pluripotency genes, indicating that observed differences in outcomes could not be explained by differences in their expression levels (Figure S6A).

Line iPSC17.2, derived from the fibroblasts of an individual with an Ex10+16 mutation in the MAPT gene, was the only line to consistently produce highly caudalized differentiations (Figure 5B). This outcome was not shared by line iPSC17.1, which

was derived from separate reprogramming of the fibroblasts from the same individual. We, therefore, performed a karyotype analysis to confirm the genomic integrity of this line and found that it had become trisomic for chromosome 12 (Figure S6B), a common event in iPSC lines (Mayshar et al., 2010), which may contribute to this outcome. All differentiations from three other lines (GMESC01.1, iPSC14.1, and iPSC12.2) also failed to generate highly cortical differentiations, though the outcomes for these lines were within the observed range for other lines (Figure 5B).

Finally, differentiation outcomes were not associated with particular disease-causing mutations or genotypes, with partly cortical differentiations (dorsalized plus partially ventralized) being generated from lines derived from control individuals (76%) and individuals affected by sporadic Alzheimer's disease (67%), Down syndrome (trisomy 21; TS21) (61%) or frontotemporal dementia due to MAPT Ex10+16 mutations (58%), though in the latter category, more of the differentiations had a partially ventralized phenotype (Figure 5C).

Patterning Variation Is Associated with Differences in Signaling Pathway Dynamics

Several signaling pathways contribute to regional patterning of the brain; most prominently, retinoic acid and the Wnt, Fgf, Hedgehog, and BMP families (Hébert and Fishell, 2008). Hedgehog signaling from the floorplate provides the main ventralizing cue of the developing forebrain (Chiang et al., 1996; Gaiano et al., 1999; Gunhaga et al., 2000). In contrast, Wnt signaling has multiple functions in the patterning of different brain regions, contingent on regionalized expression of different Wnt ligands and receptors (Montiel and Aboitiz, 2015), including promoting dorsal forebrain identities (Backman et al., 2005) and caudal identities (Nordström et al., 2002). *In vitro* patterning of human neural differentiations is also regulated by these signaling pathways, with a clear role of Hedgehog signaling in ventralization (Germain et al., 2013; Li et al., 2009; Liu et al., 2013; Maroof et al., 2013; Nicholas et al., 2013) and Wnt signaling in both dorsalization and caudalization (Elkabetz et al., 2008; Kirkeby et al., 2012; Li et al., 2009).

To investigate spontaneous variation in Wnt and Hedgehog signaling during directed differentiation, we analyzed expression of components and transcriptional readouts of both pathways in the early-stage data, grouped by differentiation outcome (dorsalized, ventralized, and caudalized). Ventralized differentiations expressed significantly higher levels of SHH and of Hedgehog signaling readouts indicative of higher pathway activation (higher PTCH1 and GLI1; lower GAS1) (Ribes and Briscoe, 2009) (Figure 5A). In contrast, Wnt signaling readouts AXIN2 and TNFRSF19 (Ha et al., 2012; Jho et al., 2002) were higher in dorsalized compared to caudalized differentiations, and in dorsalized and caudalized compared to ventralized differentiations (Figure 5A), indicating higher Wnt pathway activation in dorsalized and caudalized differentiations. Matching the cell-line-dependent frequencies of differentiation outcomes (Figure 4B), we observed variation in Hedgehog and Wnt signaling among differentiations derived from distinct PSC lines, as indicated by significant differences in SHH, GAS1, and TNFRSF19 expression (Figure 5B).

These differences in signaling cannot explain whether differential pathway activation is a cause or consequence of differences in regional identity or part of a positive-feedback loop pushing differentiations along different developmental trajectories. To determine the time when differences in signaling pathway activity begin to emerge, we collected RNA samples at 6 time points (0, 4, 7, 12, 17, and ~35 dpi) for 13 additional differentiations of 6 PSC lines chosen based on whether they were prone to generate dorsalized (iPSC21.1), partially ventralized (iPSC22.1), or highly ventralized (GMESC01.1, iPSC01.1, iPSC06.1, and iPSC14.1) differentiations (Figure 5B).

Genes accounting for the main variation along the dorsoventral axis (EMX1, EMX2, PAX6, DLX5, LHX8, and NKX2-1) had similar profiles in dorsalized and ventralized differentiations up to 12 dpi and began to diverge at 17 dpi (Figures 6C and S7A). The 17-dpi time point was also when differences in Hedgehog and Wnt signaling were first detected, with dorsalized differentiations having lower levels of Hedgehog signaling (lower PTCH1 and higher GAS1 expression) and higher levels of Wnt signaling (higher AXIN2 and TNFRSF19 expression) (Figures 6C and S7A). These results suggest that variation in culture identity and composition may arise from variation in Hedgehog and/or Wnt signaling occurring during the amplification phase (12–17 dpi).

Ventralization Is Largely Rescued by a Brief, Specific Phase of Wnt Signaling Activation

To investigate the role of Hedgehog and Wnt signaling in early patterning of differentiations, we manipulated these pathways with small-molecule inhibitors and activators during differentiation and profiled gene expression changes at ~35 dpi (Table S4). Consistent with previous reports, we found that stimulation of Hedgehog signaling using purmorphamine, a Smoothened agonist (Figure 7A), between 7 and 17 dpi caused ventralization of gene expression (Figure 7B). However, treatment with the Hedgehog signaling inhibitor cyclopamine, a Smoothened antagonist, between 7 and 17 dpi did not prevent ventralization in a spontaneously ventralizing line (Figure 7C), suggesting that endogenous Hedgehog signaling at this stage does not significantly contribute to spontaneous ventralization.

We then assessed the role of endogenous Wnt signaling in patterning by inhibiting pathway activity using the Porcupine inhibitor IWP2 (Figure 7D) between 0 and 12 dpi. The resulting differentiations had a more ventralized gene expression profile (Figure 7E), confirming that endogenous Wnt signaling is important for normal dorsalization (Li et al., 2009). Furthermore, this finding indicated that low levels of endogenous Wnt signaling may be responsible for the ventralized identity in the differentiations of our spontaneously ventralizing PSC lines. To test this hypothesis, we treated 7 differentiations from 4 ventralization-prone lines with the GSK3β inhibitor CHIR99021 (Figure 7D) between 13 and 17 dpi. When profiled at ~35 dpi, these differentiations displayed a drastic reduction in the expression of MGE-associated genes and an increase in the expression of cortical markers (Figures 7F and S7B). Furthermore, clustering of treated and untreated differentiations highlighted a shift from highly ventral clusters to more dorsalized clusters (Figure 7G). Notably, the reduction in MGE-associated genes was consistent across all 4 lines we tested (Figure 7F, bottom panels).

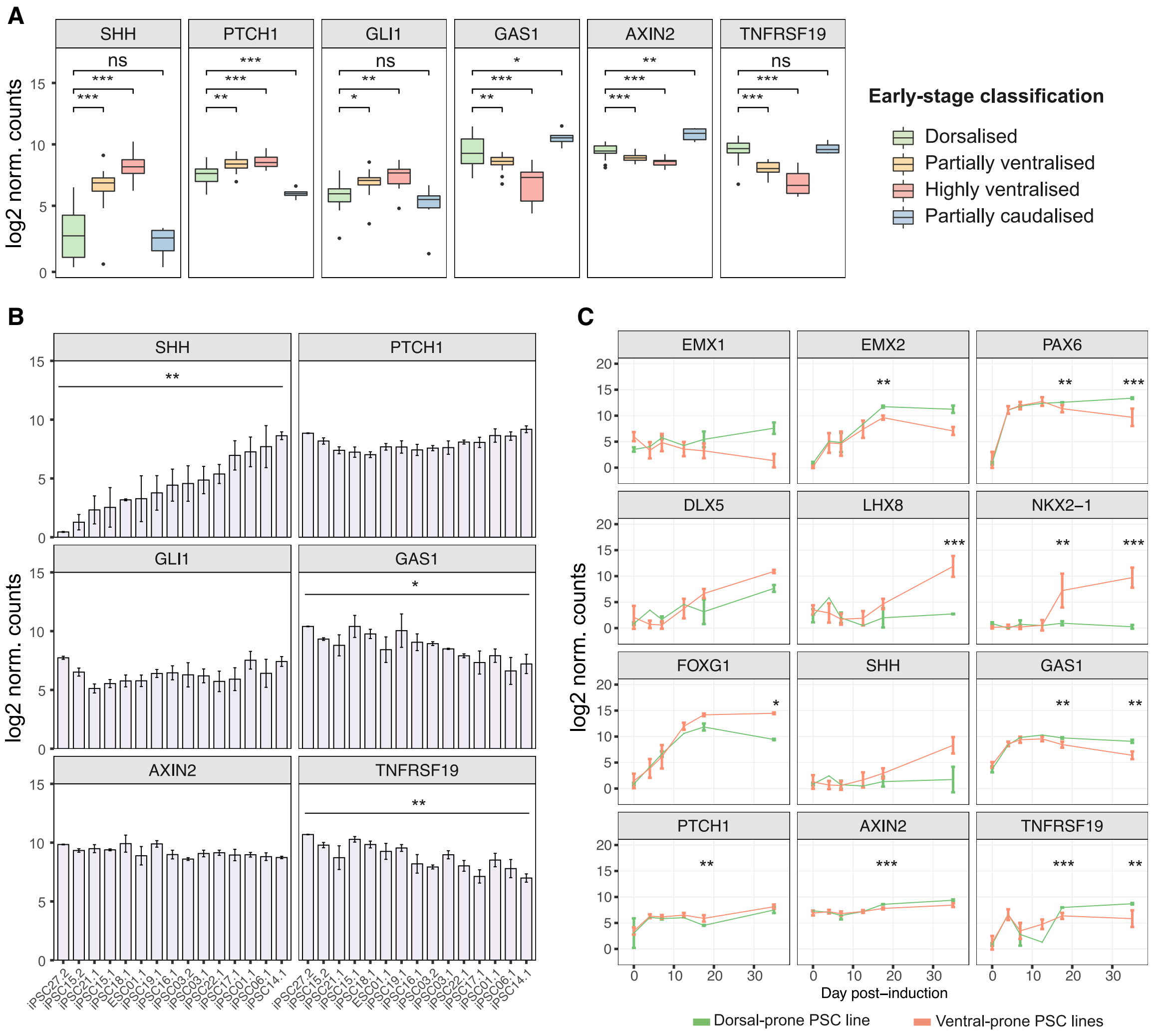

Figure 6. Hedgehog and Wnt Signaling Display Different Temporal Dynamics in PSC Lines with Inherent Tendencies to More Cortical or Ventral Differentiation Outcomes

(A) Early-stage differentiations classified as highly or partially ventralized express higher levels of SHH compared to dorsalized and caudalized differentiations, as well as levels of Hedgehog signaling readouts PTCH1, GLI1, and GAS1 consistent with increased pathway activity. Dorsalized differentiations express higher levels of Wnt signaling readouts AXIN2 and TNFRSF19 compared to ventralized differentiations but lower AXIN2 compared to partially caudalized differentiations (pairwise Welch's t test, false discovery rate [FDR]-corrected p values: $^*p < 0.05$; $^{**}p < 0.01$; $^{***}p < 0.001$; ns, not significant). Only comparisons to the dorsalized class are shown. Differentiations per group: dorsalized, 86; highly ventralized, 23; partially ventralized, 23; and partially caudalized, 14.

(B) Early-stage differentiations from separate PSC lines vary in average expression of Hedgehog and Wnt signaling pathway activation, consistent with different tendencies in regional patterning (one-way ANOVA: $^*p < 0.05$; $^{**}p < 0.01$). Error bars represent standard error; n = 2–6 differentiations per line.

(C) Gene expression time course during differentiation for selected genes associated with forebrain regions and Hedgehog or Wnt signaling. Differentiations from ventral-prone lines (partially ventral iPSC22.1 and highly ventral GMESC01.1, iPSC01.1, iPSC06.1, and iPSC14.1, n = 8–11) were compared to differentiations of a dorsal-prone line (iPSC21.1, n = 1–2). Profiles represent average gene expression, and error bars represent standard deviation. Significance shown for dorsal versus ventral comparison at 17 and ∼35 dpi (Welch's t test, FDR-corrected p values: $^*p < 0.05$; $^{**}p < 0.01$; $^{***}p < 0.001$).

Overall, these findings indicate that variability in endogenous Wnt signaling is a key contributor to variation in the regional identity acquired during directed differentiation of PSCs into cortical tissue. Stage-specific exogenous activation of Wnt signaling can promote dorsal forebrain identity when endogenous Wnt signaling is insufficient. Our results suggest that cell-line-dependent heterogeneity in Wnt pathway activation may, in part, be responsible for variation in differentiation outcomes between PSC lines (Figure 7H).

DISCUSSION

We report here that PSC-line-specific variation in endogenous Wnt signaling results in variation in composition of long-term PSC neural differentiations, with variation primarily occurring along developmental spatial axes. Our results derive from a large-scale study of 162 cortical differentiations from 61 PSC lines, which we profiled using a custom gene expression panel to investigate variation in developmental fate and cell

CellPress
OPEN ACCESS

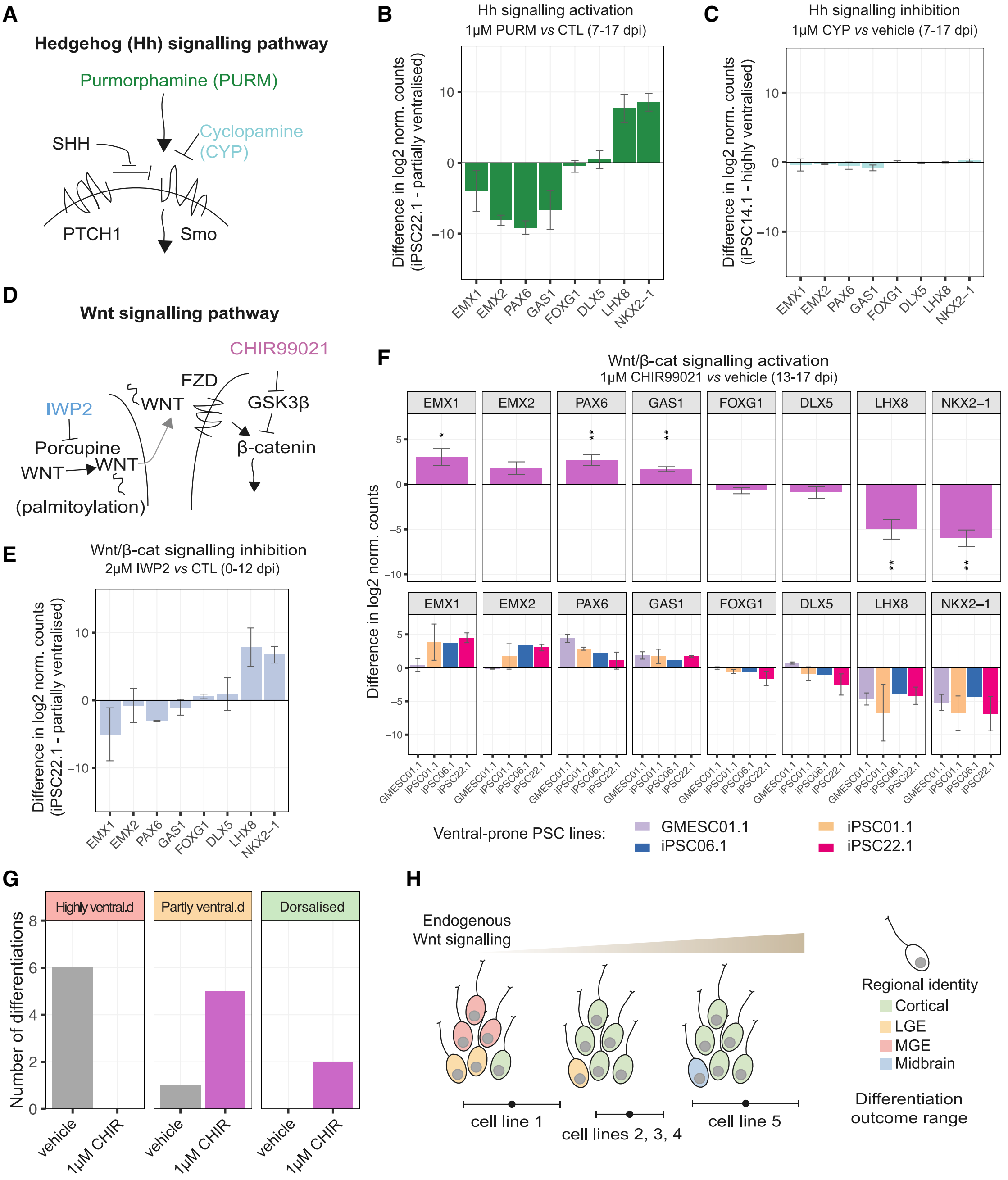

Figure 7. Inherent Tendency to Ventralization Is Largely Rescued by a Brief, Specific Phase of Wnt Signaling Activation

(A) Diagram of Hedgehog pathway components targeted by small molecules in (B) and (C).

(B) Treatment with purmorphamine (1 μM) between 7 and 17 dpi results in a more ventralized gene expression profile at 33 dpi (line iPSC22.1, n = 2).

(C) Treatment with Hedgehog inhibitor cyclopamine (1 μM) between 7 and 17 dpi has no observable effect on dorso-ventral gene expression in a highly ventralized line at ~35 dpi (iPSC14.1, n = 3).

(D) Summary diagram of Wnt pathway components targeted by small molecules in (E)–(G).

(E) Treatment with Wnt inhibitor IWP2 (2 μM) between 0 and 12 dpi results in a more ventralized gene expression profile at 33 dpi (line iPSC22.1, n = 2).

(F) Treatment with Wnt activator CHIR99021 (1 μM) between 13 and 17 dpi significantly increases cortex-associated gene expression and decreases MGE-associated expression at ~35 dpi in differentiations of 4 ventral-prone lines compared to vehicle treatment (GMESC01.1, iPSC01.1, iPSC06.1, and iPSC22.1) (one-sample Student's t test, mu = 0, n = 7, FDR-corrected p values: *$p < 0.05$; **$p < 0.01$; ***$p < 0.001$). Top panels show combined trend; bottom panels show breakdown by PSC line.

(legend continued on next page)

composition. This approach enabled us to map the variation of gene expression of *in vitro* cortical differentiations to variation along gene expression axes reflecting the dorsoventral and rostrocaudal expression axes observed during *in vivo* brain development. By assessing gene expression at two developmental stages, we confirmed the progression from neural progenitor proliferation to neurogenesis and astrogenesis; furthermore, we determined that early differences in patterning are maintained over time, indicating that early-stage assessment is predictive of late-stage fate and can be used as a quality control measure.

Variation correlating with developmental axes is strongly suggestive of a mechanism similar to the patterning mechanism of the embryonic brain, which is heavily dependent on cues from patterning centers. The Hedgehog and Wnt/β-catenin pathways have well-described roles in, respectively, providing ventralizing (Chiang et al., 1996; Gaiano et al., 1999; Gunhaga et al., 2000) and dorsalizing/caudalizing (Backman et al., 2005; Kuschel et al., 2003; McMahon and Bradley, 1990; Nordström et al., 2002; Thomas and Capecchi, 1990; Tole et al., 2000) signals during early brain development while also affecting differentiation and maturation dynamics at later times. We show here that early-stage ventralized differentiations have higher levels of Hedgehog pathway activation, that more caudalized differentiations have higher levels of Wnt signaling, and that differences in endogenous Wnt signaling are, at least in part, responsible for differences in patterning. These effects are consistent with previous studies and the signaling manipulations used in differentiation protocols for ventral forebrain and midbrain tissue (Chung et al., 2009; Elkabetz et al., 2008; Germain et al., 2013; Kirkeby et al., 2012; Li et al., 2009; Liu et al., 2013; Maroof et al., 2013). Furthermore, given the observed correlation between neuronal and ventral forebrain gene expression and the known role of the Wnt/β-catenin pathway in promoting progenitor proliferation during early cortical development (Chenn and Walsh, 2002; Hirabayashi et al., 2004; Machon et al., 2007; Woodhead et al., 2006; Wrobel et al., 2007), differences in Wnt/β-catenin signaling levels may concurrently be linked to differences in patterning and differences in the proportion of progenitors and neurons in culture.

Stochastically different activation of the Wnt/β-catenin signaling pathway among PSCs in the same neural differentiation can lead them to acquire different neural fates corresponding to neighboring brain regions (Moya et al., 2014; Yao et al., 2017). In addition, the efficiency of differentiation of a PSC line into a particular fate is influenced by variable activation of endogenous signaling pathways (Kattman et al., 2011; Nazareth et al., 2013; Nostro et al., 2011; Paige et al., 2010). We therefore hypothesize that stochastic and intrinsic differences in signaling pathway activation contribute to variation between differentiations of the same cell line and of different cell lines, respectively, so that each cell line undergoing differentiation has a range of differentiation outcomes, although usually a tight one (Figure 7H). How this might be determined by genetic and/or epigenetic factors as well as cell-culture history remains to be investigated.

Methods for directed differentiation of PSCs based on human development face similar challenges as developmental processes *in vivo*; namely, the precise canalization of cell-fate potential in spite of noisy intra- and inter-cellular signaling, genomic and epigenomic variation, and potentially variable environmental conditions. Additionally, they are only partial models of development and, thus, probably lack some of the self-regulatory mechanisms present *in vivo*. These features are likely to contribute to variation in the proportions of different cell types generated by methods aiming to produce multipotent progenitors that differentiate over a long time frame. Differences in proportion of cell types may be less problematic for studies exploiting single-cell technologies, but bulk and large-scale assays still require good quality-control measures to ensure high comparability of differentiations across cell lines and across study conditions (as well as between operators and laboratories). Implementation of such quality-control measures is likely to improve the robustness of comparisons across differentiations and thereby unlock the full potential of PSC-derived cortical differentiations, an insight that is also applicable to other differentiation protocols.

STAR★METHODS

Detailed methods are provided in the online version of this paper and include the following:

- KEY RESOURCES TABLE
- RESOURCE AVAILABILITY
  - Lead Contact
  - Materials Availability
  - Data and Code Availability
- EXPERIMENTAL MODEL AND SUBJECT DETAILS
- METHOD DETAILS
  - Reprogramming, stem cell culture and cell lines
  - Neural induction and culture
  - RNA collection
  - Genome-wide copy number assay
  - Immunostaining and quantification of cell-type proportions
  - nCounter gene expression assay and data analysis
  - Mapping to Allen Developing Mouse Brain Atlas
- QUANTIFICATION AND STATISTICAL ANALYSIS

SUPPLEMENTAL INFORMATION

Supplemental Information can be found online at https://doi.org/10.1016/j.celrep.2020.107732.

ACKNOWLEDGMENTS

We thank Prof. Tony Holland, the Dementia Research Centre, Edinburgh, Scotland, and the StemBANCC Consortium for fibroblasts or PSC lines; and

(G) Clustering of ~35-dpi differentiations from ventral-prone lines treated between 7 and 17 dpi with either vehicle or 1 μM CHIR99021. Treatment stimulating Wnt/β-catenin signaling results in shift in classificationof ventralized differentiations to more dorsalized clusters.
(H) Model for outcome of differentiation of distinct cell lines.
All error bars represent standard error. See also Figure S7.

CellPress
OPEN ACCESS

Dawei Sun, the Gurdon Institute, and Livesey lab members for sharing RNA samples. We are grateful to James Smith for technical assistance, Robert Krautz for helpful bioinformatics discussions, and Steven Moore and Philip Brownjohn for feedback on the manuscript. This work was supported by grants from Dementias Platform UK (MR/N013255/1), StemBANCC (115439), Alzheimer's Research UK (ARUK-SCRC2017-1), the Wellcome Trust (101052/Z/13/Z, 105358/Z/14/Z, and 203144), and Cancer Research UK (C6946/A24843).

AUTHOR CONTRIBUTIONS

A.S.: Conceptualization, Resources, Investigation, Data Curation, Formal Analysis, Visualization, Writing – Original Draft, Project Administration, and Funding Acquisition. E.T.: Resources, Investigation, and Writing – Review & Editing. V.E.S.: Resources, Investigation, and Writing – Review & Editing. F.J.L.: Conceptualization, Supervision, Writing – Review & Editing, Project Administration, and Funding Acquisition.

DECLARATION OF INTERESTS

The authors declare no competing interests.

Received: July 31, 2019
Revised: March 9, 2020
Accepted: May 14, 2020
Published: June 9, 2020

REFERENCES

Backman, M., Machon, O., Mygland, L., van den Bout, C.J., Zhong, W., Taketo, M.M., and Krauss, S. (2005). Effects of canonical Wnt signaling on dorso-ventral specification of the mouse telencephalon. Dev. Biol. *279*, 155–168.

Bar-Nur, O., Russ, H.A., Efrat, S., and Benvenisty, N. (2011). Epigenetic memory and preferential lineage-specific differentiation in induced pluripotent stem cells derived from human pancreatic islet beta cells. Cell Stem Cell *9*, 17–23.

Bauwens, C.L., Peerani, R., Niebruegge, S., Woodhouse, K.A., Kumacheva, E., Husain, M., and Zandstra, P.W. (2008). Control of human embryonic stem cell colony and aggregate size heterogeneity influences differentiation trajectories. Stem Cells *26*, 2300–2310.

Beers, J., Gulbranson, D.R., George, N., Siniscalchi, L.I., Jones, J., Thomson, J.A., and Chen, G. (2012). Passaging and colony expansion of human pluripotent stem cells by enzyme-free dissociation in chemically defined culture conditions. Nat. Protoc. *7*, 2029–2040.

Benjamini, Y., and Hochberg, Y. (1995). Controlling the False Discovery Rate: A Practical and Powerful Approach to Multiple Testing. J. R. Stat. Soc. B *57*, 289–300.

Carcamo-Orive, I., Hoffman, G.E., Cundiff, P., Beckmann, N.D., D'Souza, S.L., Knowles, J.W., Patel, A., Papatsenko, D., Abbasi, F., Reaven, G.M., et al. (2017). Analysis of transcriptional variability in a large human iPSC library reveals genetic and non-genetic determinants of heterogeneity. Cell Stem Cell *20*, 518–532.e9.

Chambers, S.M., Fasano, C.A., Papapetrou, E.P., Tomishima, M., Sadelain, M., and Studer, L. (2009). Highly efficient neural conversion of human ES and iPS cells by dual inhibition of SMAD signaling. Nat. Biotechnol. *27*, 275–280.

Chenn, A., and Walsh, C.A. (2002). Regulation of cerebral cortical size by control of cell cycle exit in neural precursors. Science *297*, 365–369.

Chiang, C., Litingtung, Y., Lee, E., Young, K.E., Corden, J.L., Westphal, H., and Beachy, P.A. (1996). Cyclopia and defective axial patterning in mice lacking Sonic hedgehog gene function. Nature *383*, 407–413.

Chung, S., Leung, A., Han, B.-S., Chang, M.-Y., Moon, J.-I., Kim, C.-H., Hong, S., Pruszak, J., Isacson, O., and Kim, K.-S. (2009). Wnt1-lmx1a forms a novel autoregulatory loop and controls midbrain dopaminergic differentiation synergistically with the SHH-FoxA2 pathway. Cell Stem Cell *5*, 646–658.

Clevers, H. (2016). Modeling Development and Disease with Organoids. Cell *165*, 1586–1597.

Constantin Ahlmann-Eltze (2019). ggsignif: Significance Brackets for 'ggplot2'. R package version 0.6.0. URL https://CRAN.R-project.org/package=ggsignif.

Dang, J., Tiwari, S.K., Lichinchi, G., Qin, Y., Patil, V.S., Eroshkin, A.M., and Rana, T.M. (2016). Zika Virus Depletes Neural Progenitors in Human Cerebral Organoids through Activation of the Innate Immune Receptor TLR3. Cell Stem Cell *19*, 258–265.

DeBoever, C., Li, H., Jakubosky, D., Benaglio, P., Reyna, J., Olson, K.M., Huang, H., Biggs, W., Sandoval, E., D'Antonio, M., et al. (2017). Large-Scale Profiling Reveals the Influence of Genetic Variation on Gene Expression in Human Induced Pluripotent Stem Cells. Cell Stem Cell *20*, 533–546.e7.

Elkabetz, Y., Panagiotakos, G., Al Shamy, G., Socci, N.D., Tabar, V., and Studer, L. (2008). Human ES cell-derived neural rosettes reveal a functionally distinct early neural stem cell stage. Genes Dev. *22*, 152–165.

Evseenko, D., Zhu, Y., Schenke-Layland, K., Kuo, J., Latour, B., Ge, S., Scholes, J., Dravid, G., Li, X., MacLellan, W.R., and Crooks, G.M. (2010). Mapping the first stages of mesoderm commitment during differentiation of human embryonic stem cells. Proc. Natl. Acad. Sci. USA *107*, 13742–13747.

Flames, N., Pla, R., Gelman, D.M., Rubenstein, J.L.R., Puelles, L., and Marín, O. (2007). Delineation of multiple subpallial progenitor domains by the combinatorial expression of transcriptional codes. J. Neurosci. *27*, 9682–9695.

Floruta, C.M., Du, R., Kang, H., Stein, J.L., and Weick, J.P. (2017). Default Patterning Produces Pan-cortical Glutamatergic and CGE/LGE-like GABAergic Neurons from Human Pluripotent Stem Cells. Stem Cell Reports *9*, 1463–1476.

Gaiano, N., Kohtz, J.D., Turnbull, D.H., and Fishell, G. (1999). A method for rapid gain-of-function studies in the mouse embryonic nervous system. Nat. Neurosci. *2*, 812–819.

Germain, N.D., Banda, E.C., Becker, S., Naegele, J.R., and Grabel, L.B. (2013). Derivation and isolation of NKX2.1-positive basal forebrain progenitors from human embryonic stem cells. Stem Cells Dev. *22*, 1477–1489.

Gunhaga, L., Jessell, T.M., and Edlund, T. (2000). Sonic hedgehog signaling at gastrula stages specifies ventral telencephalic cells in the chick embryo. Development *127*, 3283–3293.

Ha, A., Perez-Iratxeta, C., Liu, H., Mears, A.J., and Wallace, V.A. (2012). Identification of Wnt/β-catenin modulated genes in the developing retina. Mol. Vis. *18*, 645–656.

Hébert, J.M., and Fishell, G. (2008). The genetics of early telencephalon patterning: some assembly required. Nat. Rev. Neurosci. *9*, 678–685.

Hirabayashi, Y., Itoh, Y., Tabata, H., Nakajima, K., Akiyama, T., Masuyama, N., and Gotoh, Y. (2004). The Wnt/beta-catenin pathway directs neuronal differentiation of cortical neural precursor cells. Development *131*, 2791–2801.

Hu, B.-Y., Weick, J.P., Yu, J., Ma, L.-X., Zhang, X.-Q., Thomson, J.A., and Zhang, S.-C. (2010). Neural differentiation of human induced pluripotent stem cells follows developmental principles but with variable potency. Proc. Natl. Acad. Sci. USA *107*, 4335–4340.

Jho, E.H., Zhang, T., Domon, C., Joo, C.-K., Freund, J.-N., and Costantini, F. (2002). Wnt/beta-catenin/Tcf signaling induces the transcription of Axin2, a negative regulator of the signaling pathway. Mol. Cell. Biol. *22*, 1172–1183.

Kajiwara, M., Aoi, T., Okita, K., Takahashi, R., Inoue, H., Takayama, N., Endo, H., Eto, K., Toguchida, J., Uemoto, S., and Yamanaka, S. (2012). Donor-dependent variations in hepatic differentiation from human-induced pluripotent stem cells. Proc. Natl. Acad. Sci. USA *109*, 12538–12543.

Kattman, S.J., Witty, A.D., Gagliardi, M., Dubois, N.C., Niapour, M., Hotta, A., Ellis, J., and Keller, G. (2011). Stage-specific optimization of activin/nodal and BMP signaling promotes cardiac differentiation of mouse and human pluripotent stem cell lines. Cell Stem Cell *8*, 228–240.

Kelava, I., and Lancaster, M.A. (2016). Stem cell models of human brain development. Cell Stem Cell *18*, 736–748.

Kilpinen, H., Goncalves, A., Leha, A., Afzal, V., Alasoo, K., Ashford, S., Bala, S., Bensaddek, D., Casale, F.P., Culley, O.J., et al. (2017). Common genetic variation drives molecular heterogeneity in human iPSCs. Nature *546*, 370–375.

Kim, K., Doi, A., Wen, B., Ng, K., Zhao, R., Cahan, P., Kim, J., Aryee, M.J., Ji, H., Ehrlich, L.I., et al. (2010). Epigenetic memory in induced pluripotent stem cells. Nature *467*, 285–290.

Kirkeby, A., Grealish, S., Wolf, D.A., Nelander, J., Wood, J., Lundblad, M., Lindvall, O., and Parmar, M. (2012). Generation of regionally specified neural progenitors and functional neurons from human embryonic stem cells under defined conditions. Cell Rep. *1*, 703–714.

Kobayashi, T., Mizuno, H., Imayoshi, I., Furusawa, C., Shirahige, K., and Kageyama, R. (2009). The cyclic gene Hes1 contributes to diverse differentiation responses of embryonic stem cells. Genes Dev. *23*, 1870–1875.

Kuschel, S., Rüther, U., and Theil, T. (2003). A disrupted balance between Bmp/Wnt and Fgf signaling underlies the ventralization of the Gli3 mutant telencephalon. Dev. Biol. *260*, 484–495.

Li, X.-J., Zhang, X., Johnson, M.A., Wang, Z.-B., Lavaute, T., and Zhang, S.-C. (2009). Coordination of sonic hedgehog and Wnt signaling determines ventral and dorsal telencephalic neuron types from human embryonic stem cells. Development *136*, 4055–4063.

Liu, Y., Liu, H., Sauvey, C., Yao, L., Zarnowska, E.D., and Zhang, S.-C. (2013). Directed differentiation of forebrain GABA interneurons from human pluripotent stem cells. Nat. Protoc. *8*, 1670–1679.

Machon, O., Backman, M., Machonova, O., Kozmik, Z., Vacik, T., Andersen, L., and Krauss, S. (2007). A dynamic gradient of Wnt signaling controls initiation of neurogenesis in the mammalian cortex and cellular specification in the hippocampus. Dev. Biol. *311*, 223–237.

Maroof, A.M., Keros, S., Tyson, J.A., Ying, S.-W., Ganat, Y.M., Merkle, F.T., Liu, B., Goulburn, A., Stanley, E.G., Elefanty, A.G., et al. (2013). Directed differentiation and functional maturation of cortical interneurons from human embryonic stem cells. Cell Stem Cell *12*, 559–572.

Mayshar, Y., Ben-David, U., Lavon, N., Biancotti, J.-C., Yakir, B., Clark, A.T., Plath, K., Lowry, W.E., and Benvenisty, N. (2010). Identification and classification of chromosomal aberrations in human induced pluripotent stem cells. Cell Stem Cell *7*, 521–531.

McMahon, A.P., and Bradley, A. (1990). The Wnt-1 (int-1) proto-oncogene is required for development of a large region of the mouse brain. Cell *62*, 1073–1085.

Menendez, L., Kulik, M.J., Page, A.T., Park, S.S., Lauderdale, J.D., Cunningham, M.L., and Dalton, S. (2013). Directed differentiation of human pluripotent cells to neural crest stem cells. Nat. Protoc. *8*, 203–212.

Merkle, F.T., Maroof, A., Wataya, T., Sasai, Y., Studer, L., Eggan, K., and Schier, A.F. (2015). Generation of neuropeptidergic hypothalamic neurons from human pluripotent stem cells. Development *142*, 633–643.

Molyneaux, B.J., Arlotta, P., Menezes, J.R.L., and Macklis, J.D. (2007). Neuronal subtype specification in the cerebral cortex. Nat. Rev. Neurosci. *8*, 427–437.

Montiel, J.F., and Aboitiz, F. (2015). Pallial patterning and the origin of the isocortex. Front. Neurosci. *9*, 377.

Moore, S., Evans, L.D.B., Andersson, T., Portelius, E., Smith, J., Dias, T.B., Saurat, N., McGlade, A., Kirwan, P., Blennow, K., et al. (2015). APP metabolism regulates tau proteostasis in human cerebral cortex neurons. Cell Rep. *11*, 689–696.

Mora-Bermúdez, F., Badsha, F., Kanton, S., Camp, J.G., Vernot, B., Köhler, K., Voigt, B., Okita, K., Maricic, T., He, Z., et al. (2016). Differences and similarities between human and chimpanzee neural progenitors during cerebral cortex development. eLife *5*, e18683.

Mormone, E., D'Sousa, S., Alexeeva, V., Bederson, M.M., and Germano, I.M. (2014). "Footprint-free" human induced pluripotent stem cell-derived astrocytes for in vivo cell-based therapy. Stem Cells Dev. *23*, 2626–2636.

Moya, N., Cutts, J., Gaasterland, T., Willert, K., and Brafman, D.A. (2014). Endogenous WNT signaling regulates hPSC-derived neural progenitor cell heterogeneity and specifies their regional identity. Stem Cell Reports *3*, 1015–1028.

Najm, F.J., Zaremba, A., Caprariello, A.V., Nayak, S., Freundt, E.C., Scacheri, P.C., Miller, R.H., and Tesar, P.J. (2011). Rapid and robust generation of functional oligodendrocyte progenitor cells from epiblast stem cells. Nat. Methods *8*, 957–962.

Nazareth, E.J.P., Ostblom, J.E.E., Lücker, P.B., Shukla, S., Alvarez, M.M., Oh, S.K.W., Yin, T., and Zandstra, P.W. (2013). High-throughput fingerprinting of human pluripotent stem cell fate responses and lineage bias. Nat. Methods *10*, 1225–1231.

Neuwirth, E. (2014). RColorBrewer: ColorBrewer Palettes. R package version 1.1-2. URL https://CRAN.R-project.org/package=RColorBrewer.

Nicholas, C.R., Chen, J., Tang, Y., Southwell, D.G., Chalmers, N., Vogt, D., Arnold, C.M., Chen, Y.-J.J., Stanley, E.G., Elefanty, A.G., et al. (2013). Functional maturation of hPSC-derived forebrain interneurons requires an extended timeline and mimics human neural development. Cell Stem Cell *12*, 573–586.

Nordström, U., Jessell, T.M., and Edlund, T. (2002). Progressive induction of caudal neural character by graded Wnt signaling. Nat. Neurosci. *5*, 525–532.

Nostro, M.C., Sarangi, F., Ogawa, S., Holtzinger, A., Corneo, B., Li, X., Micallef, S.J., Park, I.-H., Basford, C., Wheeler, M.B., et al. (2011). Stage-specific signaling through TGFβ family members and WNT regulates patterning and pancreatic specification of human pluripotent stem cells. Development *138*, 861–871.

Osafune, K., Caron, L., Borowiak, M., Martinez, R.J., Fitz-Gerald, C.S., Sato, Y., Cowan, C.A., Chien, K.R., and Melton, D.A. (2008). Marked differences in differentiation propensity among human embryonic stem cell lines. Nat. Biotechnol. *26*, 313–315.

Otani, T., Marchetto, M.C., Gage, F.H., Simons, B.D., and Livesey, F.J. (2016). 2D and 3D stem cell models of primate cortical development identify species-specific differences in progenitor behavior contributing to brain size. Cell Stem Cell *18*, 467–480.

Paige, S.L., Osugi, T., Afanasiev, O.K., Pabon, L., Reinecke, H., and Murry, C.E. (2010). Endogenous Wnt/β-catenin signaling is required for cardiac differentiation in human embryonic stem cells. PLoS ONE *5*, e11134.

Qian, X., Nguyen, H.N., Song, M.M., Hadiono, C., Ogden, S.C., Hammack, C., Yao, B., Hamersky, G.R., Jacob, F., Zhong, C., et al. (2016). Brain-Region-Specific Organoids Using Mini-bioreactors for Modeling ZIKV Exposure. Cell *165*, 1238–1254.

Raivo Kolde (2019). pheatmap: Pretty Heatmaps. R package version 1.0.12. URL https://CRAN.R-project.org/package=pheatmap.

R Core Team (2019). R: A language and environment for statistical computing. R Foundation for Statistical Computing, Vienna, Austria. URL https://www.R-project.org/.

Ribes, V., and Briscoe, J. (2009). Establishing and interpreting graded Sonic Hedgehog signaling during vertebrate neural tube patterning: the role of negative feedback. Cold Spring Harb. Perspect. Biol. *1*, a002014.

Santagati, F., and Rijli, F.M. (2003). Cranial neural crest and the building of the vertebrate head. Nat. Rev. Neurosci. *4*, 806–818.

Shaltouki, A., Peng, J., Liu, Q., Rao, M.S., and Zeng, X. (2013). Efficient generation of astrocytes from human pluripotent stem cells in defined conditions. Stem Cells *31*, 941–952.

Shi, Y., Inoue, H., Wu, J.C., and Yamanaka, S. (2017). Induced pluripotent stem cell technology: a decade of progress. Nat. Rev. Drug Discov. *16*, 115–130.

Shi, Y., Kirwan, P., and Livesey, F.J. (2012b). Directed differentiation of human pluripotent stem cells to cerebral cortex neurons and neural networks. Nat. Protoc. *7*, 1836–1846.

Shi, Y., Kirwan, P., Smith, J., MacLean, G., Orkin, S.H., and Livesey, F.J. (2012a). A human stem cell model of early Alzheimer's disease pathology in Down syndrome. Sci. Transl. Med. *4*, 124ra29.

Shi, Y., Kirwan, P., Smith, J., Robinson, H.P.C., and Livesey, F.J. (2012c). Human cerebral cortex development from pluripotent stem cells to functional excitatory synapses. Nat. Neurosci. *15*, 477–486, S1.

Siller, R., Naumovska, E., Mathapati, S., Lycke, M., Greenhough, S., and Sullivan, G.J. (2016). Development of a rapid screen for the endodermal differentiation potential of human pluripotent stem cell lines. Sci. Rep. *6*, 37178.

CellPress
OPEN ACCESS

Simunovic, M., and Brivanlou, A.H. (2017). Embryoids, organoids and gastruloids: new approaches to understanding embryogenesis. Development *144*, 976–985.

Sussel, L., Marin, O., Kimura, S., and Rubenstein, J.L. (1999). Loss of Nkx2.1 homeobox gene function results in a ventral to dorsal molecular respecification within the basal telencephalon: evidence for a transformation of the pallidum into the striatum. Development *126*, 3359–3370.

Teo, A.K.K., Ali, Y., Wong, K.Y., Chipperfield, H., Sadasivam, A., Poobalan, Y., Tan, E.K., Wang, S.T., Abraham, S., Tsuneyoshi, N., et al. (2012). Activin and BMP4 synergistically promote formation of definitive endoderm in human embryonic stem cells. Stem Cells *30*, 631–642.

Thomas, K.R., and Capecchi, M.R. (1990). Targeted disruption of the murine int-1 proto-oncogene resulting in severe abnormalities in midbrain and cerebellar development. Nature *346*, 847–850.

Tole, S., Ragsdale, C.W., and Grove, E.A. (2000). Dorsoventral patterning of the telencephalon is disrupted in the mouse mutant extra-toes(J). Dev. Biol. *217*, 254–265.

Tsankov, A.M., Akopian, V., Pop, R., Chetty, S., Gifford, C.A., Daheron, L., Tsankova, N.M., and Meissner, A. (2015). A qPCR ScoreCard quantifies the differentiation potential of human pluripotent stem cells. Nat. Biotechnol. *33*, 1182–1192.

van de Leemput, J., Boles, N.C., Kiehl, T.R., Corneo, B., Lederman, P., Menon, V., Lee, C., Martinez, R.A., Levi, B.P., Thompson, C.L., et al. (2014). CORTECON: a temporal transcriptome analysis of in vitro human cerebral cortex development from human embryonic stem cells. Neuron *83*, 51–68.

Volpato, V., Smith, J., Sandor, C., Ried, J.S., Baud, A., Handel, A., Newey, S.E., Wessely, F., Attar, M., Whiteley, E., et al. (2018). Reproducibility of Molecular Phenotypes after Long-Term Differentiation to Human iPSC-Derived Neurons: A Multi-Site Omics Study. Stem Cell Reports *11*, 897–911.

Whitfield, M.L., George, L.K., Grant, G.D., and Perou, C.M. (2006). Common markers of proliferation. Nat. Rev. Cancer *6*, 99–106.

Wickham, H. (2007). Reshaping Data with the reshape Package. J. Stat. Softw. *21*, 1–20.

Wickham, H. (2011). The Split-Apply-Combine Strategy for Data Analysis. J. Stat. Softw. *40*, 1–29.

Wickham, H. (2016). ggplot2: Elegant Graphics for Data Analysis (Springer).

Wickham, H., and Henry, L. (2020). tidyr: Tidy Messy Data. R package version 1.0.3. URL https://CRAN.R-project.org/package=tidyr

Woodhead, G.J., Mutch, C.A., Olson, E.C., and Chenn, A. (2006). Cell-autonomous β-catenin signaling regulates cortical precursor proliferation. J. Neurosci. *26*, 12620–12630.

Wrobel, C.N., Mutch, C.A., Swaminathan, S., Taketo, M.M., and Chenn, A. (2007). Persistent expression of stabilized beta-catenin delays maturation of radial glial cells into intermediate progenitors. Dev. Biol. *309*, 285–297.

Yao, Z., Mich, J.K., Ku, S., Menon, V., Krostag, A.-R., Martinez, R.A., Furchtgott, L., Mulholland, H., Bort, S., Fuqua, M.A., et al. (2017). A Single-Cell Roadmap of Lineage Bifurcation in Human ESC Models of Embryonic Brain Development. Cell Stem Cell *20*, 120–134.

Yasunaga, M., Tada, S., Torikai-Nishikawa, S., Nakano, Y., Okada, M., Jakt, L.M., Nishikawa, S., Chiba, T., Era, T., and Nishikawa, S. (2005). Induction and monitoring of definitive and visceral endoderm differentiation of mouse ES cells. Nat. Biotechnol. *23*, 1542–1550.

Zhou, T., Tan, L., Cederquist, G.Y., Fan, Y., Hartley, B.J., Mukherjee, S., Tomishima, M., Brennand, K.J., Zhang, Q., Schwartz, R.E., et al. (2017). High-Content Screening in hPSC-Neural Progenitors Identifies Drug Candidates that Inhibit Zika Virus Infection in Fetal-like Organoids and Adult Brain. Cell Stem Cell *21*, 274–283.e5.

STAR★METHODS

KEY RESOURCES TABLE

REAGENT or RESOURCE	SOURCE	IDENTIFIER
Antibodies		
Anti-TBR1	Abcam	Cat# ab31940; RRID:AB_2200219
Anti-CTIP2	Abcam	Cat# ab18465; RRID:AB_2064130
Anti-TBR2	Abcam	Cat# ab23345; RRID:AB_778267
Anti-GABA	Sigma-Aldrich	Cat# A2052; RRID:AB_477652
Anti-SOX2	Abcam	Cat# ab79351; RRID:AB_10710406
Biological Samples		
Foetal Lung RNA	Laboratory of Emma Rawlins	N/A
Chemicals, Peptides, and Recombinant Proteins		
ROCK inhibitor (Y-27632)	Tocris Bioscience	Cat# 1254
SB431542	Tocris Bioscience	Cat# 1614
Dorsomorphin	Tocris Bioscience	Cat# 3093
LDN193189	Cell Guidance Systems	Cat# SM23
FGF2	Cambridge Stem Cell Institute	N/A
Purmorphamine	Tocris Bioscience	Cat# 4551
Cyclopamine	StemCell Technologies	Cat# 72072
CHIR99021	Sigma-Aldrich	Cat# SML1046
IWP2	StemCell Technologies	Cat# 72122
Critical Commercial Assays		
nCounter Gene Expression assays	This paper; NanoString Technologies	Table S1
Qubit RNA BR assay kit	ThermoFisher Scientific	Cat# Q10210
Qubit RNA HS assay kit	ThermoFisher Scientific	Cat# Q32855
CytoTune®-iPS 2.0 Sendai Reprogramming Kit	ThermoFisher Scientific	Cat# A16517
RNeasy mini kit	QIAGEN	Cat# 74106
DNeasy Blood & Tissue kit	QIAGEN	Cat# 69506
Infinium HumanCytoSNP-12	Illumina	Cat# WG-320-2101
CytoSNP850K	Illumina	Cat# 20025643
Deposited Data		
Normalized gene expression data	This paper	Tables S3 & S4
Mouse brain *in vivo* expression energy data	Allen Developing Mouse Brain Atlas, Allen Institute for Brain Science	https://developingmouse.brain-map.org/
ScRNA-seq of cortical iPSC differentiations	Yao et al., 2017; GEO repository	GSE86977
Experimental Models: Cell Lines		
See Table S2 for full info on 61 PSC lines		
Software and Algorithms		
R v 3.6.2	R Core Team, 2019	https://www.R-project.org/
R package - dplyr	dplyr: A Grammar of Data Manipulation. R package version 0.8.5.	https://cran.r-project.org/web/packages/dplyr/index.html
R package - ggplot2	Wickham (2016). ggplot2: Elegant Graphics for Data Analysis. Springer-Verlag New York.	https://ggplot2.tidyverse.org
R package - ggsignif	Ahlmann-Eltze, 2019	https://cran.r-project.org/web/packages/ggsignif/index.html
R package - pheatmap	Kolde, 2019	https://cran.r-project.org/web/packages/pheatmap/index.html

(*Continued on next page*)

Continued

REAGENT or RESOURCE	SOURCE	IDENTIFIER
R package - RColorBrewer	Neuwirth, 2014	https://cran.r-project.org/web/packages/RColorBrewer/index.html
R package - reshape2	Wickham (2007). Reshaping Data with the reshape Package. Journal of Statistical Software, 21(12), 1-20.	https://www.jstatsoft.org/v21/i12/
R package - plyr	Wickham (2011). The Split-Apply-Combine Strategy for Data Analysis. Journal of Statistical Software, 40(1), 1-29.	https://www.jstatsoft.org/v40/i01/
R package – tidyr	Wickham and Henry, 2020	https://cran.r-project.org/web/packages/tidyr/index.html
nSolver software –v 3.0	NanoString Technologies	https://www.nanostring.com/products/analysis-software/nsolver
Harmony High-Content Imaging and Analysis Software	Perkin Elmer	https://www.perkinelmer.com/product/harmony-4-9-office-license-hh17000010

RESOURCE AVAILABILITY

Lead Contact

Further information and requests for resources and reagents should be directed to Prof. Rick Livesey (r.livesey@ucl.ac.uk).

Materials Availability

In this study 31 iPSC lines were generated in house from fibroblasts (see Table S2). There are restrictions to the distribution of the cell lines due to resources involved in line expansion, maintenance and storage. We will share cell lines with reasonable compensation by the requestor for its processing and shipping but we may require a completed Materials Transfer Agreement.

Data and Code Availability

The published article includes the datasets generated and analyzed in this study (Tables S3 and S4).

In situ hybridization images used in Figures S2A and S3B and gene expression data used for mapping of *in vitro* differentiations to *in vivo* developmental regions in Figures 2E and 3E were obtained from the Allen Developing Mouse Brain Atlas © 2008 Allen Institute for Brain Science. Available from: https://developingmouse.brain-map.org/. Expression energy data for probes in the gene expression panels were downloaded using the Allen Brain Atlas API (http://help.brain-map.org/display/devmouse/API).

Single-cell RNA-sequencing data used in Figure S3C was generated by Yao et al., 2017 and accessed through the GEO repository (GSE86977).

EXPERIMENTAL MODEL AND SUBJECT DETAILS

Details on genotype, sex, and cell type of origin for all cell lines used are provided in Table S2.

All PSC lines were cultured in E8 medium (Thermo Fisher Scientific, A1517001) on Geltrex (Thermo Fisher Scientific, A1413301) as previously described (Beers et al., 2012).

Selected cell lines were authenticated using SNP data to validate line identity, genomic integrity (or expected trisomy 21 for cell lines derived from individuals with Down syndrome), and sex.

METHOD DETAILS

Reprogramming, stem cell culture and cell lines

All PSC line information is provided in Table S2. Of the 61 lines used in this study, 13 were previously published, 10 were reprogrammed and characterized by the STEMBANCC consortium, and 36 were reprogrammed in-house from fibroblasts using the integration-free CytoTune®-iPS 2.0 Sendai Reprogramming Kit (Life Technologies) as per manufacturer's instructions. Twenty-one days after viral infection individual iPSC-like colonies were manually picked for expansion into individual iPSC lines and transferred onto feeder-free Geltrex-coated plates and cultured with Essential 8 medium (Life Technologies). Elimination of Sendai vectors was confirmed by RT-PCR at passage > 10. All PSC lines were cultured in E8 medium on Geltrex as described (Beers et al., 2012). Briefly, when confluence reached ~80%, PSCs colonies were washed rapidly with 0.5 mM EDTA, dissociated with 0.5 mM EDTA for 2-4 minutes, resuspended in E8 medium and re-plated at a variable split ratio (~1:6) on Geltrex-coated plates. Pluripotency gene expression was measured using custom nCounter gene expression codesets (Codeset1, Codeset3 – Table S1). Data analysis

was performed as described in the *nCounter gene expression assay and data analysis* section after merging the datasets using the MultiRLF Merge function of the nSolver software.

Neural induction and culture

Neural induction was performed based on a published protocol (Shi et al., 2012b) with a few modifications. Briefly, human ESCs or iPSCs were dissociated to single cells with Accutase (Sigma) and plated at ~260,000 cells/cm^2 in E8 with 10 μM ROCK inhibitor (Y-27632, Tocris Bioscience) on tissue culture plates coated with Geltrex (Thermo Fisher A1413302). They were allowed to attach for at least six hours to overnight, washed once with PBS, and then the medium was replaced with neural induction medium (NIM) consisting of a 1:1 mix of N2 (N2 supplement in DMEM/F12, Thermo Fisher 17502048 and 31331093) and B27 (B27 supplement in Neurobasal, Thermo Fisher 17504044 and 12348017) supplemented with 10 μM SB431542 and 1 μM Dorsomorphin (Tocris Bioscience) (Day 0). For some inductions Dorsomorphin was substituted with 100 nM LDN193189 (Cell Guidance Systems). NIM was replaced daily for 12 days; on day 12, the neuroepithelial sheet was detached from the plate using Dispase II (Thermo Fisher 17105) and replated on plates coated with laminin (Sigma L2020). From the following day until day 17 cultures were grown in N2B27 supplemented with 10-20 ng/ul FGF2, replaced daily or on alternate days, and from day 17 onward in N2B27 medium only. Between day 17 and 25 any non-neural differentiation present was removed by passaging with Dispase as required, and the neural cultures were then dissociated to single cells using Accutase. When cultures reached ~80%–90% confluence they were passaged again until a final passage between day 33-38, when they were plated for long-term culture, after which N2B27 medium was replaced every second day. For experiments manipulating signaling pathways, the induction or maintenance medium were supplemented with 1 μM purmorphamine (Tocris Bioscience), 1 μM cyclopamine (StemCell technologies), 1 μM CHIR99021 (Sigma), or 2 μM IWP2 (StemCell Technologies) during the indicated time window (Figures 6 & S7) and replaced daily.

RNA collection

All RNA samples from cortical differentiations and PSCs were collected by adding RLT lysis buffer directly to the rinsed culture plate or to a collected pellet of dissociated cultures, and RNA was then extracted using the QIAGEN RNeasy spin columns. RNA from 20 pcw human fetal lung was a kind gift from Dawei Sun and Emma Rawlins (Gurdon Institute, Cambridge).

Genome-wide copy number assay

Genomic DNA from the indicated PSC lines was extracted using the QIAGEN DNeasy Blood & Tissue kit and analyzed on the Illumina Infinium HumanCytoSNP-12 (iPSC17.1-2) or CytoSNP850K (iPSC22.1) platforms.

Immunostaining and quantification of cell-type proportions

The same 34-dpi differentiations were plated contemporarily on Geltrex-coated CellCarrier Ultra 96-well plates at ~75,000 cells/cm^2 and on Geltrex-coated 24-well plates at ~65,000 cells/cm^2. We plated 36 cultures from 29 separate differentiations of 12 different PSC lines (1-3 differentiations per line; 6 differentiations plated in duplicate). Each culture was assigned a code and operator was blind to differentiation identity when collecting RNA expression and imaging data. After 21 days RNA was collected from the 24-well plates and cultures in the 96-well plates were fixed using 4% PFA in PBS for 10 min. To calculate correlation between gene expression and differentiation composition, we chose optimized antibodies against antigens with quantifiable nuclear expression. Immunostaining was performed using primary antibodies against TBR1 (Abcam ab31940, 1:250), CTIP2 (Abcam ab18465, 1:500), TBR2/EOMES (Abcam ab23345, 1:250), GABA (Sigma-Aldrich A2052, 1:1,000), and SOX2 (Abcam ab79351, 1:200) and using Alexa-conjugated secondary antibodies. Normal donkey serum (5%) in TBS with 0.3% Triton-X was used as blocking solution. Automatic confocal imaging was performed on an Opera Phoenix High Content Screening System (Perkin Elmer, HH14000000) and 85 fields of view were acquired per culture. Nuclei segmentation was performed on the DAPI channel using Method C within the Opera Harmony software (Common Threshold: 0.1; Volume > 60 μm^3; Splitting Coefficient:7; Individual Threshold:0.45; Contrast > 0.1). Antigen-positive nuclei were determined based on intensity comparison to control unstained with primary antibodies. The percentage of antigen-positive nuclei was calculated over the total number of counted nuclei in each differentiation (average: 69,437, range: 18,229-89,160). Samples in which fewer than 10,000 nuclei were detected, indicative of widespread detachment, were discarded. The final dataset included data on 16 cultures from 15 differentiations of 8 PSC lines (1-3 differentiations per line, 1 differentiation in duplicate); detachment preferentially affected highly ventral differentiations. The numbers of cultures, differentiations, and lines used to calculate each correlation in Figure S1E are as follows: TBR1 (15/14/7), CTIP2 (15/14/7), TBR2 (16/15/8), GABA (15/14/8) and SOX2 (16/15/8).

nCounter gene expression assay and data analysis

Gene expression was profiled using custom-designed nCounter gene expression codesets on the nCounter® SPRINT Profiler platform. Samples in the study were run with one of two codesets containing respectively 200 and 156 gene probes (Codeset1 and 2, Table S1). RNA concentration was measured using Qubit RNA Assay kits (ThermoFisher) or by spectrophotometric analysis using a Hidex Sense instrument, and 50 ng total RNA were loaded for each nCounter assay. Each differentiation was assigned a unique induction number (UIN). Transcript counts were normalized on the nSolver analysis software or in R by subtracting the geometric mean of 8 negative control probes, and by multiplying by two sample-specific normalization factors obtained using the geometric mean of 6 positive control probes, and the geometric mean of 7 housekeeping genes (CLTC, GAPDH, GUSB, PPIA, RPLP1, RPS15A, RPS9).

Samples with a positive control or housekeeping normalization factor larger than 4 or smaller than 0.25 were removed and the remaining samples were re-normalized without outliers.

Gene expression data used to study variation between differentiation are reported in Table S3. The number of samples in the final datasets was 84 for early-stage Codeset1, 44 for late-stage Codeset1, 65 for early-stage Codeset2, and 13 for late-stage Codeset2. To prevent any codeset effects on variation, analyses in Figures 1–3 and S1, S2, S3, and S4 were performed on the two codesets separately (excluding late-stage Codeset2, which included fewer than 15 samples). Figure 6A and 6B only contain samples from the early-stage, Codeset2 dataset due to lack of the probes of interest in Codeset1. For classification purposes (Figure 4) we combined the two datasets from the same time window using the MultiRLF Merge function of the nSolver software (Table S3), using 5 samples that were profiled with both codesets as cross-codeset normalizers, resulting in a combined dataset of 149 early-stage and 57 late-stage samples (Table S3), including 44 differentiations with paired data at both temporal stages. Gene expression data used to study temporal dynamics (Figures 6C and S7A) and the effect of signaling pathway manipulations (Figures 7 and S7B) are reported in Table S4.

For all datasets, the probe list was filtered to include only genes whose expression was above 30 normalized counts in at least one of the samples. We chose this threshold based on our assessment of technical noise and to be above the average level of the negative control probe with the highest counts (Figures S1A and S1B). All analyses were performed in R v3.6.2 (R Core Team, 2019) on log-2 transformed data to decrease skewness and normalize variance. Pearson correlation was used to calculate gene expression correlations and sample clustering distances using the cor function and pheatmap package, while principal component analysis was performed using the prcomp function. Additionally, the dplyr, plyr, tidyr, and reshape2 packages were used for data manipulation and the ggplot2, ggsignif, and RColorBrewer packages were used for data visualization.

Mapping to Allen Developing Mouse Brain Atlas

Expression energy data at E11.5 for genes present in our Nanostring panels were downloaded using the Allen Institute API. To cover most of the brain, regions from the ventral telencephalon to the hindbrain were considered: the rostral secondary prosencephalon (RSP), pallium, subpallium, diencephalon, peduncular hypothalamus, midbrain, and hindbrain. Since expression of most genes was correlated across regions, variable genes expressed selectively in particular regions were identified as those with large (> 2) regression residuals in each pairwise comparison with other regions. No genes in the panel could be identified as selective for diencephalon and peduncular hypothalamus therefore these regions were removed from the set. Repeating the analysis identified 3-10 genes selectively expressed in each region compared to others for a total of 27 genes (RSP: GPC3, FOXP2, PTCH1, SATB2, RAX; subpallium: LHX6, OLIG2, DLX1, LHX8, GSC; pallium: MAP2, FOXG1, TBR1, SLC17A7, GLI3, EOMES, GAS1, EMX1, NEUROG2, EMX2; midbrain: DDC, NR4A2, OTX2, EN2; hindbrain: HOXB2, NKX6-1, HOXA2). Using expression of this subset of genes, Spearman correlation was used to calculate a mapping score of each differentiation to each region of the E11.5 mouse brain and scores for each differentiation were then z-normalized. Mapping of late-stage differentiations was similarly performed using E15.5 expression data, where 19 selectively expressed genes were identified (RSP: RAX, GPC3, PTGDS, OTP; subpallium: GAD2, LHX8, LHX6; pallium: NEUROG2, NEUROD2, NEUROD1, NFIX, FEZF2, SATB2, SOX5; midbrain: OTX2, POU4F2; hindbrain: NKX6-1, TFAP2A, HOXA2).

QUANTIFICATION AND STATISTICAL ANALYSIS

The statistical tests used, the definitions of center, dispersion, and precision, and the number of samples (n) and their identity is indicated in each figure legend. To compare temporal dynamics (Figure 6C), we first averaged multiple samples from the same unique untreated or vehicle-treated differentiations and then compared the values for all unique differentiations from ventral-prone lines to those from the dorsal-prone line. Means of multiple differentiations per line are shown in Figure S7A. To assess the effect of the pharmacological interventions (Figure 7), we compared gene expression values from samples of drug-treated differentiations to those from the same differentiations when untreated (purmorphamine, IWP2) or vehicle-treated (cyclopamine [ethanol], CHIR99021 [DMSO]) over the same time-window, including where vehicle treatment length exceeded drug treatment length. For both analyses, samples were pooled into time points: ~17.5 (range 17-18) and ~35 (range 30-39).

All statistical analyses were performed in R v3.6.2 (R Core Team, 2019). Welch's t tests were performed as unpaired, two-sided tests with function t.test without assuming equal variance (var.equal = FALSE). One-way ANOVA was performed using the function aov. One-sample Student t tests were performed as two-sided tests with function t.test, setting mu = 0. No tests were performed to assess normality assumption. P value correction for multiple testing was performed using the p.adjust function using the FDR (Benjamini and Hochberg, 1995) correction.

Cell Reports, Volume *31*

Supplemental Information

Variable Outcomes in Neural Differentiation of Human PSCs Arise from Intrinsic Differences in Developmental Signaling Pathways

Alessio Strano, Eleanor Tuck, Victoria E. Stubbs, and Frederick J. Livesey

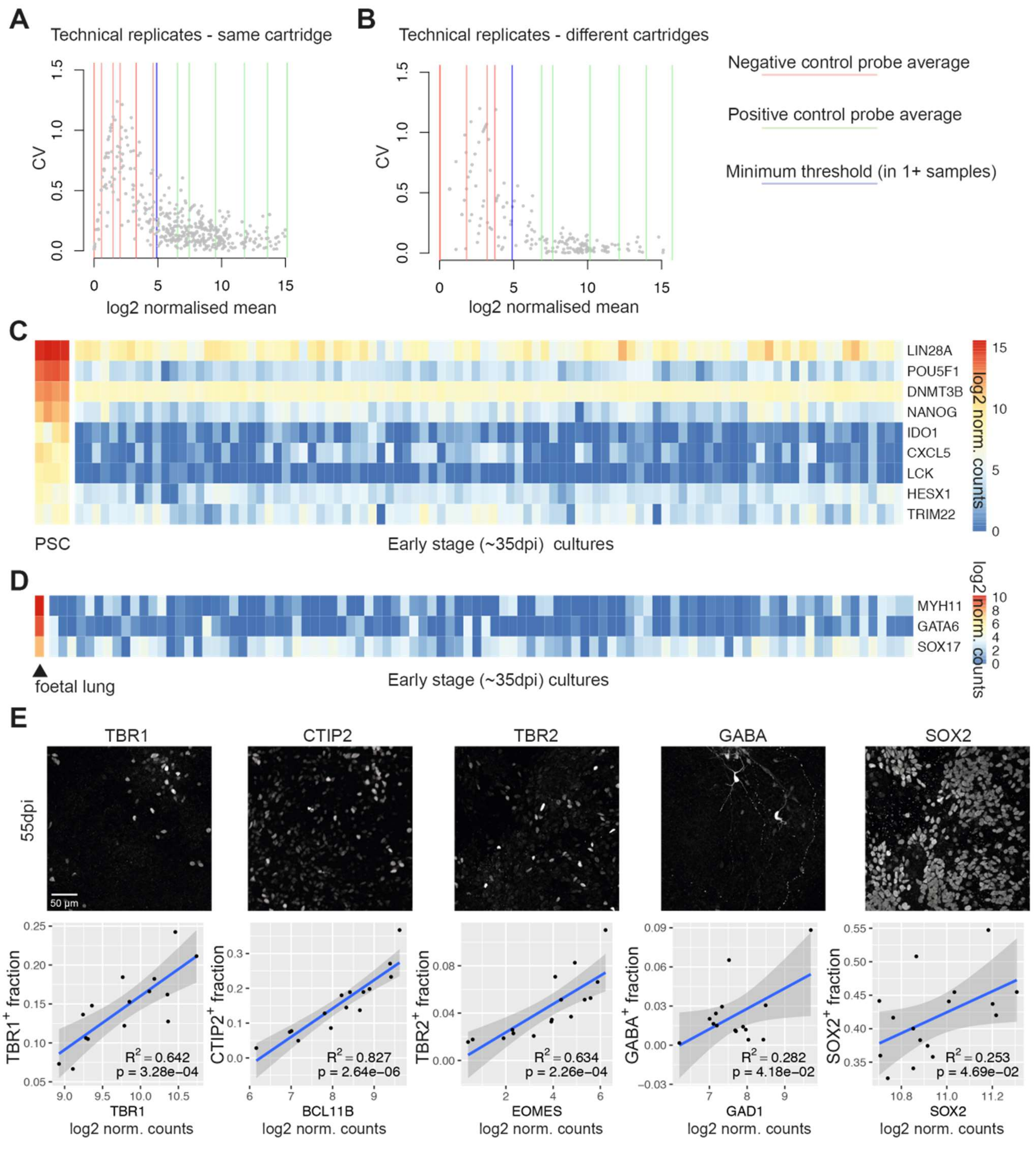

Figure S1. **Gene expression panel technical and biological validation.** Related to Figure 1.

(A-B) Relationship between mean gene expression and coefficient of variation in 3 technical replicates (3 samples each) run on the same cartridge (A) and 2 technical replicates run on different cartridges and 9 months apart (B). Dots represent individual gene probes; red and green vertical lines represent respectively the average for the negative and positive control probes included in the codeset. The blue vertical line represents the minimum threshold of expression we required in at least one sample to include a gene probe in any analysis.

(C) Heatmap of expression of PSC genes showing highly reduced or absent expression in 84 early-stage cortical differentiations compared to 4 PSC samples.

(D) Heatmap of expression of genes expressed in mesodermal and endodermal tissue showing no expression in 84 early-stage cortical differentiations compared to a 20pcw human foetal lung sample. (E) Correlations between expression of selected genes and the fraction of cells immunopositive for the corresponding protein (TBR1, CTIP2, TBR2, SOX2) or enzymatic product (GABA).

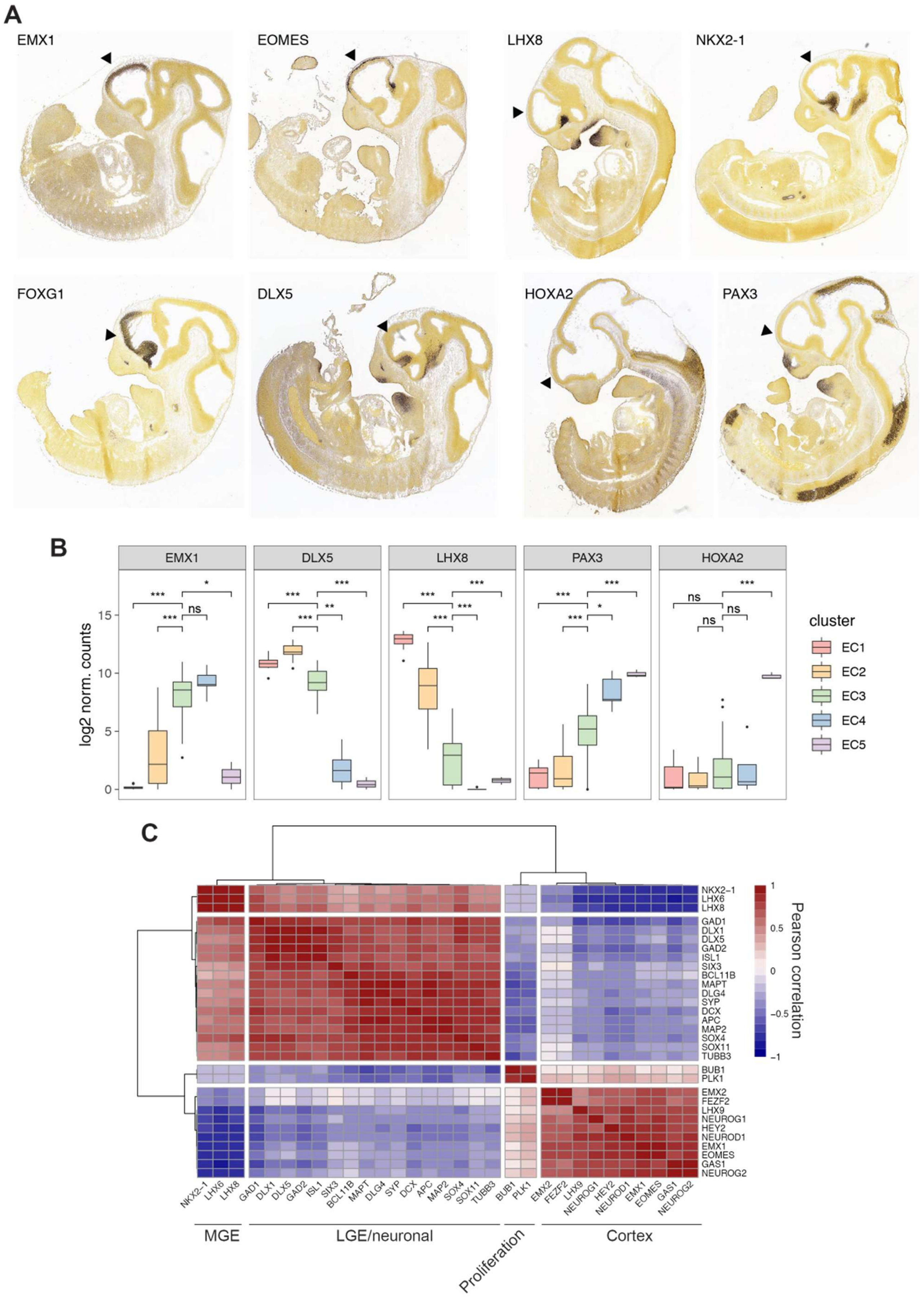

A
EMX1
EOMES
LHX8
NKX2-1
FOXG1
DLX5
HOXA2
PAX3
B
EMX1
DLX5
LHX8
PAX3
HOXA2
log2 norm. counts
cluster
EC1
EC2
EC3
EC4
EC5
C
MGE
LGE/neuronal
Proliferation
Cortex
Pearson correlation
NKX2−1
LHX6
LHX8
GAD1
DLX1
DLX5
GAD2
ISL1
SIX3
BCL11B
MAPT
DLG4
SYP
DCX
APC
MAP2
SOX4
SOX11
TUBB3
BUB1
PLK1
EMX2
FEZF2
LHX9
NEUROG1
HEY2
NEUROD1
EMX1
EOMES
GAS1
NEUROG2

Figure S2. Largest contributors to early-stage variation are enriched in specific brain regions during embryonic development. Related to Figure 2.

(A) Expression of selected high-loading genes measured by in-situ hybridisation in E11.5 mouse embryos (from the Allen Developing Mouse Brain Atlas © 2008 Allen Institute for Brain Science). A black arrowhead marks the developing cortex.

(B) Differences in expression of selected high-loading genes among the groups determined in Figure 2A (pairwise Welch's t-test, FDR corrected p-values: <0.05 (*), <0.01 (**), <0.001 (***), ns (not significant); only comparisons to EC3 are shown). Differentiations per group: EC1 (10), EC2 (28), EC3 (38), EC4 (5), EC5 (3).

(C) Heatmap of Pearson correlation coefficients for early-stage gene expression (n =84). Only genes with an absolute correlation coefficient greater than 0.85 for at least one other gene are included.

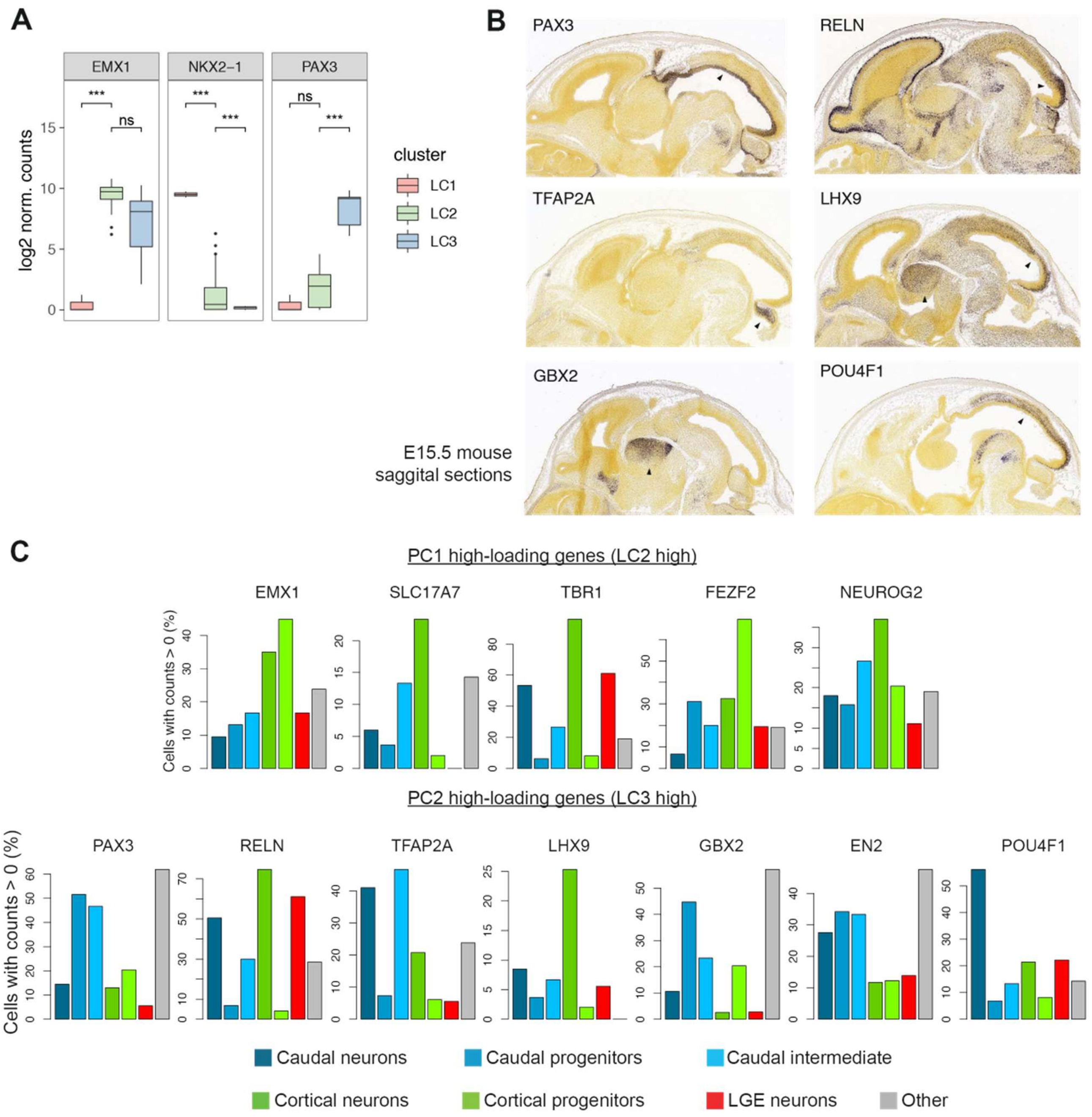

Figure S3. **Largest contributors to late-stage variation along PC2 are enriched in cell types with caudal identity.** Related to Figure 3.

(A) Differences in expression of selected high-loading genes among the groups determined in Figure 3A (pairwise Welch's t-test, FDR corrected p-values: <0.05 (*), <0.01 (**), <0.001 (***), ns (not significant)). Differentiations per group: LC1 (3), LC2 (36), LC3 (5).

(B) Expression of selected high-loading genes measured by in-situ hybridisation in E15.5 mouse embryos (from the Allen Developing Mouse Brain Atlas © 2008 Allen Institute for Brain Science).

(C) Expression of selected high-loading genes in scRNA-seq data from 54-dpi forebrain differentiations from Yao et al., 2016. For each gene, the percentage of single cells with at least one transcript count is plotted for cell populations of different lineages. Genes associated with LC1 are more highly expressed in progenitors and neurons of cortical lineage,

while most genes associated with LC2 are highly expressed in caudal progenitors and undetermined cell type (PAX3, GBX2, EN2), and in caudal neuronal types (TFAP2A, POU4F1).

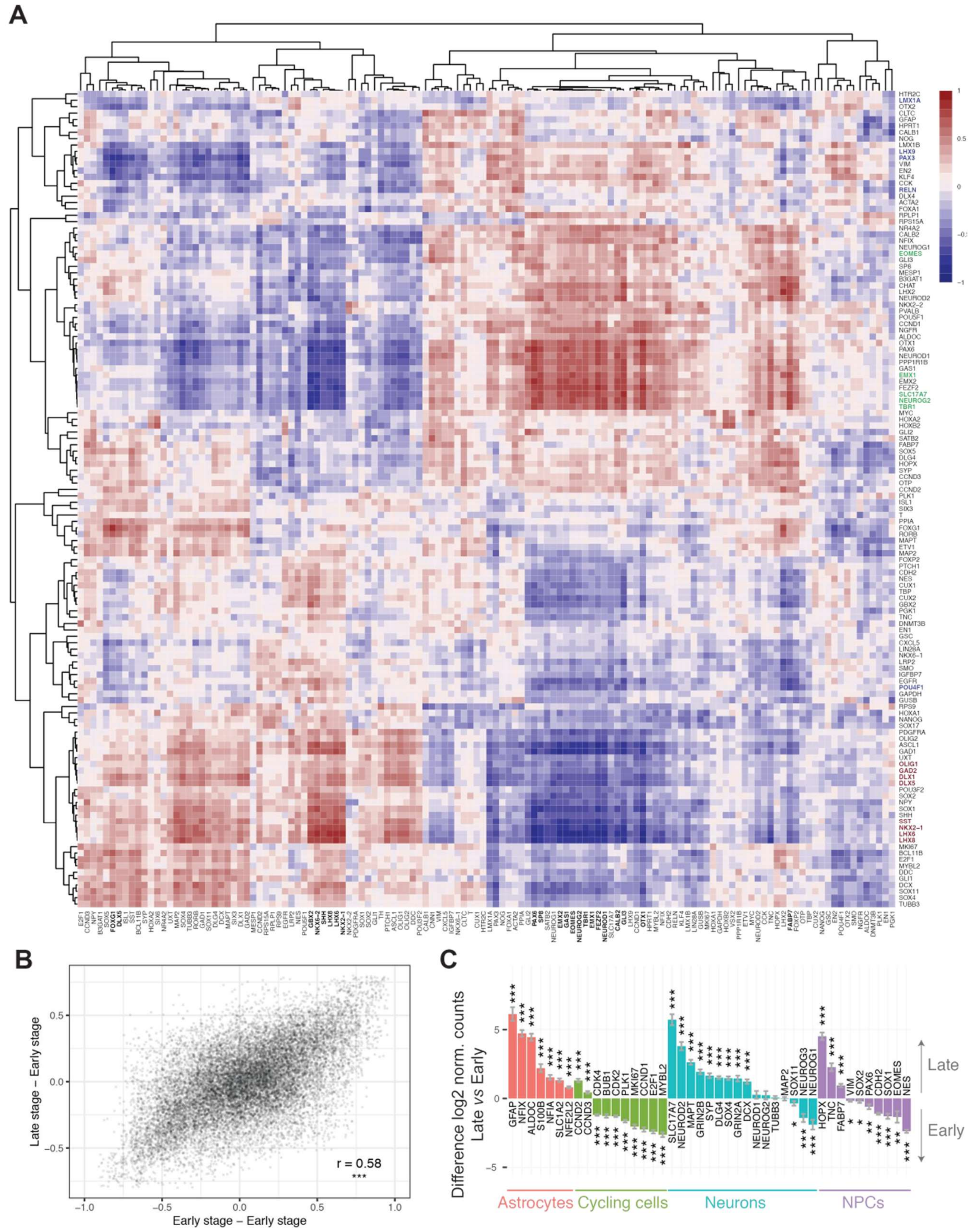

Figure S4. Late-stage expression reflects developmental temporal progression. Related to Figure 4.

(A) Pearson correlation between gene expression in late-stage differentiations (y-axis) and gene expression in early-stage differentiations (x-axis) for 44 time pairs. Late-stage cluster markers are reported in bold on the y-axis and colour coded

in green (dorsalised - LC2), red (ventralised - LC1), and blue (partially caudalised – LC3). Early-stage genes with an absolute correlation coefficient greater than 0.75 with any of these are indicated in bold on the x-axis, and selectively shown in Figure 4A.

(B) Relationship between expression correlation of any gene pair in the early-stage dataset and between the early-stage and late-stage dataset. The significant correlation between these two datasets indicates that expression of gene modules present at the early-stage is maintained at the late-stage, and therefore early-stage expression of these modules can predict late-stage expression.

(C) Average difference in expression between late-stage (~ 82 days post induction, dpi) and early-stage (~35 dpi) dorsalised differentiations. The expression of most astrocytic and neuronal genes increases, while most NPC and proliferation genes decrease over time (one-sample t-test on difference in paired late *vs* early samples, n = 30 pairs, mu = 0; FDR corrected p-values: <0.05 (*), <0.01 (**), <0.001 (***)). Error bars represent standard error.

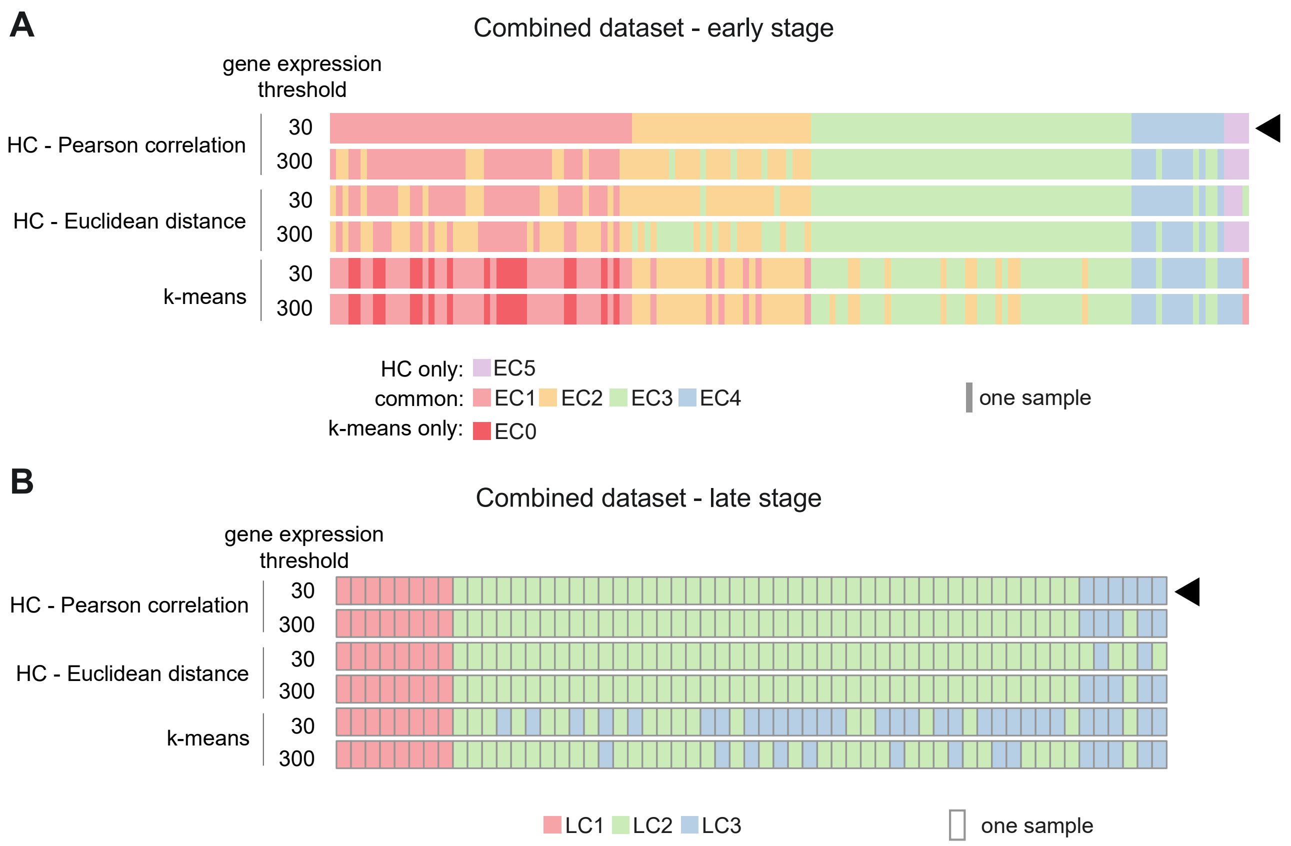

Figure S5. **Cluster similarity with different clustering approaches.** Related to Figure 4.

Separate rows represent cluster assignments for the combined early-stage dataset (A, 149 samples) or late-stage dataset (B, 57 samples) using indicated clustering method (k-means or HC: hierarchical clustering), clustering distance (Pearson correlation or Euclidean distance), and minimum threshold in at least one sample (30 or 300 normalised counts). Arrowheads indicate clustering used in the main text.

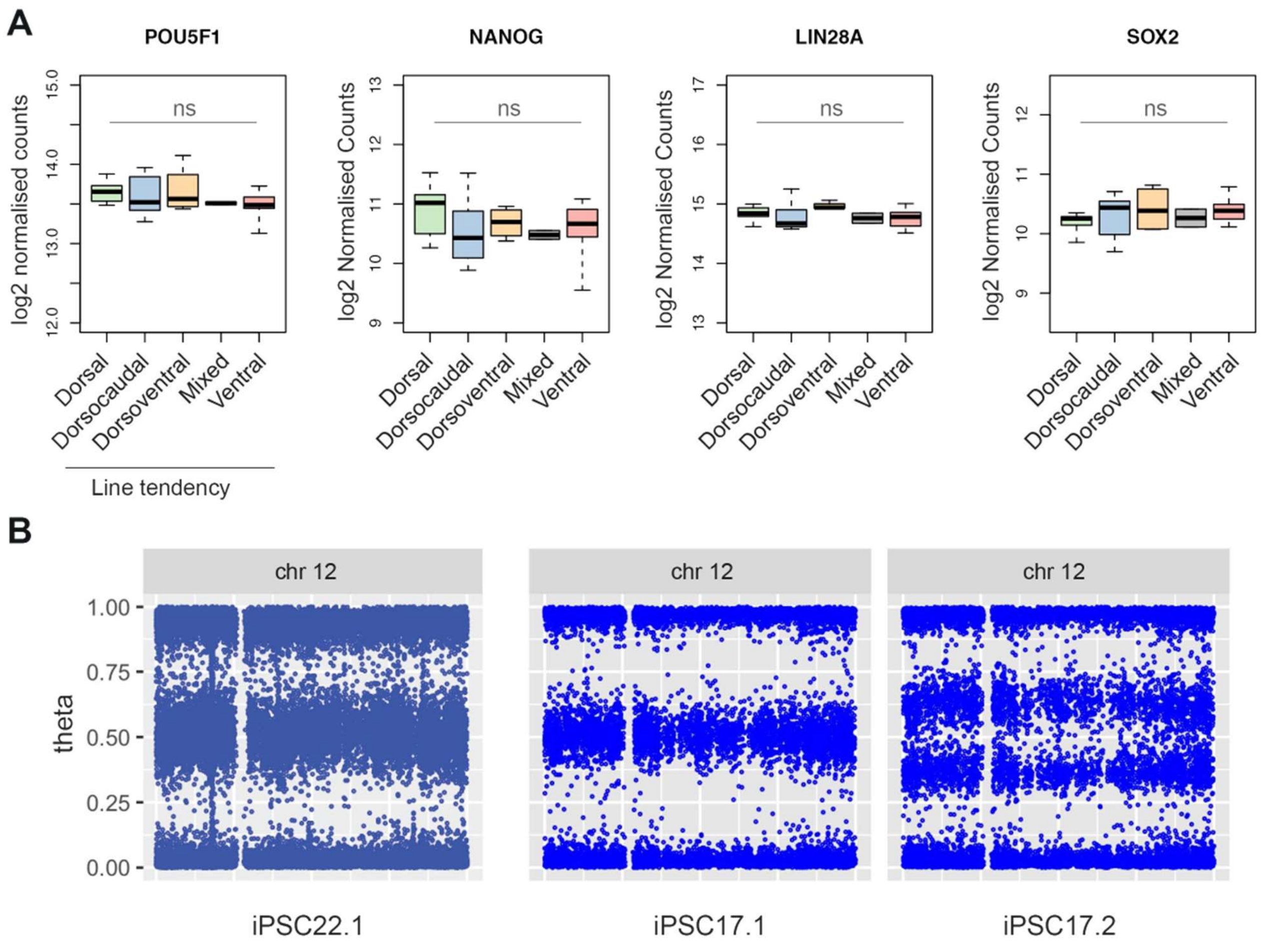

Figure S6. **Cell line contributions to differentiation outcome.** Related to Figure 5.

(A) Expression levels of key pluripotency genes grouped by patterning-proneness of the corresponding PSC line (one-way ANOVA, not significant). PSC lines per group: dorsal (7), dorsocaudal (5), dorsoventral (4), mixed (2), ventral (8).

(B) Allelic SNP ratio (theta) for single nucleotide polymorphism loci on chromosome 12 for a control line (iPSC22.1) and two clones derived from the same individual carrying a MAPT Ex10+16 mutation (iPSC17.1-2). The horizontal axis represents normalised chromosomal distance. The presence of 4 bands in iPSC17.2 indicates trisomy of chromosome 12.

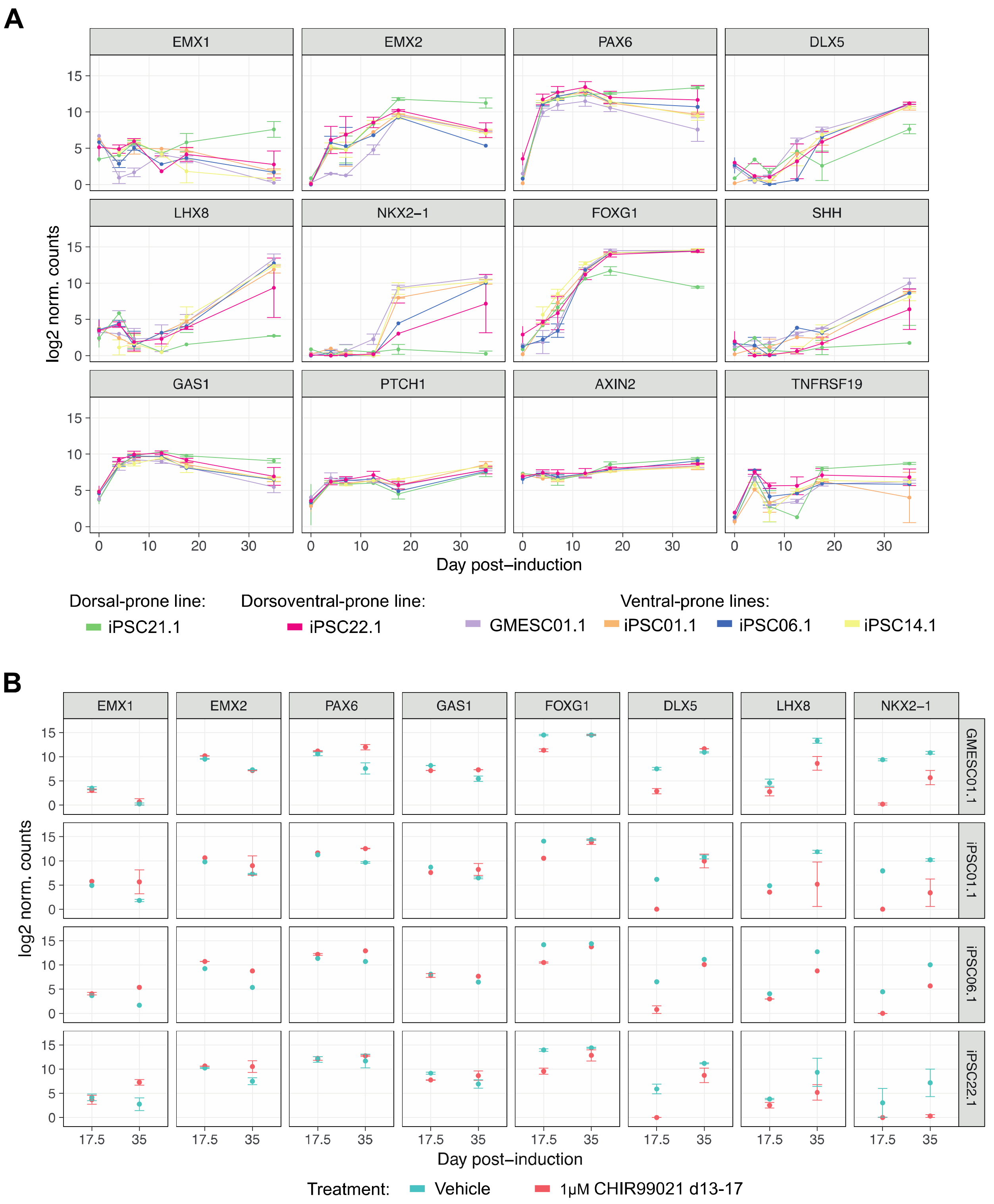

Figure S7. CHIR99021 treatment dorsalises gene expression in spontaneously-ventralising differentiations. Related to Figures 6 and 7.

(A) Gene expression timecourse during differentiation for selected genes associated with forebrain regions and Hedgehog or Wnt signalling pathways. Profiles from six PSC lines are shown, each profile represents the average of 1-3 differentiations. Notice the graded differences between a dorsal-prone line (iPSC21.1), a dorsoventral-prone line

(iPSC22.1), and four ventral-prone lines (GMESC01.1, iPSC01.1, iPSC06.1, iPSC14.1). Profiles represent average gene expression per line and error bars represent standard deviation.

(B) Log2 normalised count data corresponding to Figure 7F. Expression of key markers at ~17 and ~35 dpi is shown for four dorsoventral/ventral-prone lines treated with either vehicle or Wnt signalling activator CHIR99021 (1 μM) between 13-17 dpi. Number of differentiations (n = 2) except for iPSC01.1 at 17dpi and vehicle treated iPSC06.1, for which a single data point is shown. Error bars represent standard error.

SUPPLEMENTARY TABLES (see separate files)

Table S1. Nanostring Codesets Probe Sequences, Related to Figure 1

Table S2. Details of PSC Lines Used and their Differentiation Trends, Related to Figure 5

Table S3. Normalised Gene Expression Matrices for Assessment of Variation, Related to Figures 1-6

Table S4. Normalised Gene Expression Matrices for Temporal Dynamics and Effects of Signalling Manipulation, Related to Figures 6-7
